# Phylogeography of *Parasyncalathium souliei* (Asteraceae) and Its Potential Application in Delimiting Phylogeoregions in the Qinghai-Tibet Plateau (QTP)-Hengduan Mountains (HDM) Hotspot

**DOI:** 10.3389/fgene.2018.00171

**Published:** 2018-05-17

**Authors:** Nan Lin, Tao Deng, Michael J. Moore, Yanxia Sun, Xianhan Huang, Wenguang Sun, Dong Luo, Hengchang Wang, Jianwen Zhang, Hang Sun

**Affiliations:** ^1^Key Laboratory of Plant Germplasm Enhancement and Specialty Agriculture, Wuhan Botanical Garden, Chinese Academy of Sciences, Wuhan, China; ^2^University of Chinese Academy of Sciences, Beijing, China; ^3^Key Laboratory for Plant Diversity and Biogeography of East Asia, Kunming Institute of Botany, Chinese Academy of Sciences, Kunming, China; ^4^Department of Biology, Oberlin College, Oberlin, OH, United States

**Keywords:** Qinghai-Tibet Plateau (QTP)-Hengduan Mountains (HDM), *Parasyncalathium souliei*, phylogeography, genetic distribution, phylogeoregion

## Abstract

Biogeographic regionalization can help to better understand diversity in biogeography, conservation, and macroecology. Historical regionalization schemes typically focus on species distributions, often rarely considering the rich context that phylogeographic information can provide. We investigated whether phylogeographic data could help to delineate floristic regions in the Qinghai-Tibet Plateau (QTP)-Hengduan Mountains (HDM) region by analyzing phylogeographic structure in the herb *Parasyncalathium souliei* (Asteraceae). We sequenced the plastid *psbA*-*trnH* and *trnL-rpl32* spacer regions for 417 individuals in 36 populations across the geographic range of the species. To estimate the phylogeographic history of this species, a series of population genetic, phylogenetic, molecular dating, and haplotype network analyses were conducted, as were tested for historical demographic expansions. Using occurrence data, species distribution modeling was used to estimate geographic distributions at three time points: the present, the Mid-Holocene and the Last Glacial Maximum. Significant phylogeographic structure was evident (*N*_*ST*_> *G*_*ST*_; *P* < 0.05) among the 37 haplotypes detected. Four major haplogroups were identified based on phylogenetic analyses. Private haplotypes were restricted to geographically distinct regions that generally corresponded to previously identified biogeographic subregions within the QTP-HDM region. Our results imply Pliocene-Pleistocene diversification of *P. souliei* and suggest that the species may have been geographically widespread early in its history. This study may provide valuable evidence for phylogeographic regionalization using chloroplast genetic data in a common, widespread endemic species from the QTP-HDM.

## Introduction

**Figure 1:**
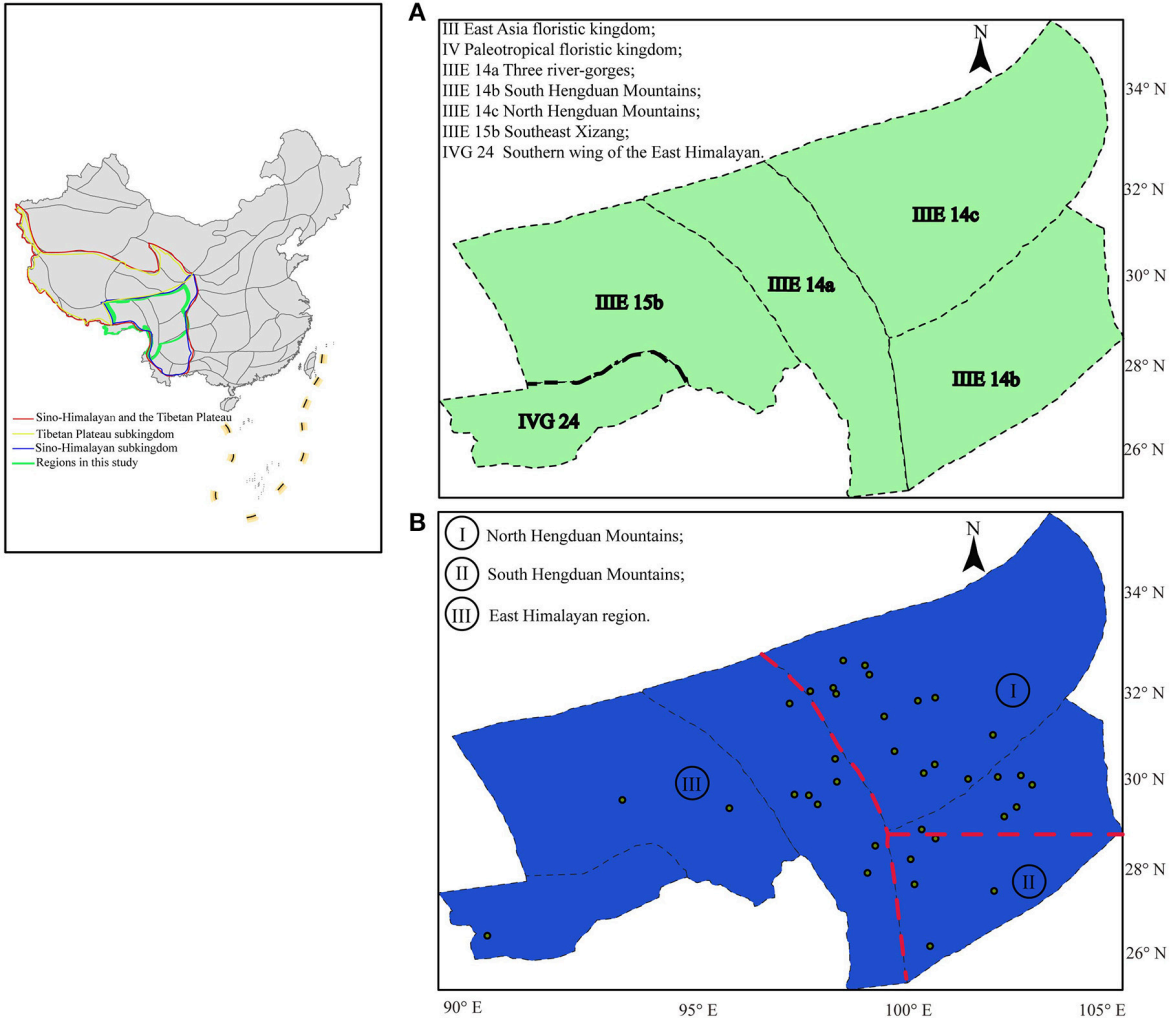
Biogeographic regions proposed for the flora of the QTP-HDM: **(A)** Previous regions proposed and modified from Wu and Wu (1996); **(B)** Simplified phylogeoregions proposed in this study based on private haplotypes: phylogeoregions I, II, and III represent the north and south Hengduan Mountains phylogeoregions, and the Eastern Himalayan phylogeoregion, respectively. The dots in **(B)** correspond to the 36 natural populations of *Parasyncalathium souliei* in this study.

The Himalayas, especially their core regions, i.e., the Qinghai-Tibet Plateau (QTP) and the Hengduan Mountains (HDM), comprise one of the key high-altitude biodiversity hotspots in the world (Myers et al., 2000). Geological and climatic differences have created profound ecological heterogeneity in the QTP and HDM: eastern regions have deep valleys characterized mainly by a warm and wet climate, whereas the high-elevation central and western Himalayas are characterized by a cold and dry climate (Wu and Wu, 1996; Favre et al., 2015). In an influential study, Wu and Wu (1996) divided these regions into two floristic subkingdoms, Sino-Himalayan and the Tibetan Plateau, based on the distribution of taxa endemic to these areas (Figure 1A). The former subkingdom includes most parts of the Yunnan Plateau, HDM, and the East Himalayan region, while the latter subkingdom is composed of most regions of the QTP including the Tangut the Tibet–Pamir–Kunlun, and the Western Himalayan region. Wu and Wu (1996) further divided the center of the HDM region itself into three subregions: the northern, southern, and Three river-gorges regions (Wu and Wu, 1996; Figure 1A). In the past two decades phylogeographic studies have been conducted in the QTP-HDM region (see introduction of Ren et al., 2017). Almost all of these studies have demonstrated that past climatic changes, including at least some related to geological events, have affected the demographic history of the organisms and have further suggested that multiple plant refugia probably existed in the Himalayas (Sun et al., 2017; Chen et al., 2018). Nevertheless, the results of these studies have rarely been incorporated into analyses of phylogeographic regionalization, in contrast to other areas such as South America (Amarilla et al., 2015).

*Parasyncalathium souliei* J. W. Zhang, Boufford and H. Sun (Asteraceae: Cichorieae) (Zhang et al., 2011a) is a widespread species of the QTP-HDM that represents an excellent opportunity to explore the phylogeography of this region. As the only species in the recently described genus *Parasyncalathium, P. souliei* is known from five of the QTP-HDM subregions of Wu and Wu (1996): the North Hengduan Mountains (hereafter called NHDM), the South Hengduan Mountains (hereafter called SHDM), Three river-gorges, Southeast Xizang and Southern wing of the East Himalayan (Figure 1A; Wu and Wu, 1996). Previous biogeographic studies have suggested that this species may have originated relatively recently (Zhang et al., 2011b; Kilian et al., 2017). *P*. *souliei* is small, biennial rosette-forming herb up to 19 cm tall that occurs in well-delimited patches within open bare ground (Zhang et al., 2011b). Heads are congested and are surrounded by a ring of basal leaves, and fruits are likely wind-dispersed (Zhang et al., 2011b). Individuals are relatively small and have a short life span, with relatively high reproductive and mutation rates (Robert et al., 2013). *P*. *souliei* is a pioneer species in the QTP-HDM, and thus particularly sensitive to environmental/habitat change (Zhang et al., 2011b).

In this study, we employed an integrative approach to identify phylogeographic patterns within *P. souliei* and to determine which factors are responsible for its current geographic distribution. We also propose the concept “phylogeoregion” to delimit regions with phylogeographically concordant patterns. More specifically, our goals were to explore: (1) how topography/geography and Pleistocene climatic oscillations have shaped the present-day distributions of *P. souliei*; (2) whether significant divergence and genetic structure exists among *P. souliei* populations across different floristic regions/subregions; and (3) whether phylogeographic information may be useful in characterizing potential phylogeoregions in the QTP-HDM region.

## Materials and methods

### DNA samples, PCR, and sequencing

All the Material was collected and deposited in the Herbarium of the Kunming Institute of Botany, China. Otherwise, no specific permissions were required for material collection of the nature reserves about sampling and investigate in field of Provincial Forestry Department; the locations of *Parasyncalathium souliei* are not privately-owned and are endangered or protected in the field. A total of 417 individuals representing 36 populations of *P. souliei* were collected from across the geographic range of the species. Within each population, 3–19 individuals growing at least 40 m apart were collected to maximize sampling of genetic diversity within populations. For most populations, all individuals found were sampled to obtain maximum coverage of *P. souliei*. Fresh leaves of all individuals were dried in the field using silica gel. Total genomic DNA was extracted using a QIAGEN DNeasy Plant Mini Kit (QIAGEN, Beijing, China). Two plastid DNA spacer regions (*psbA*-*trnH* and *trnL-rpl32*) were amplified via PCR in 80 μl reactions, each containing 30–40 ng of template DNAs, 50 mM Tris-HCl, 1.5 mM MgCl_2_, 0.5 mM dNTPs, 2 mM of each primer and 0.75 U of Taq polymerase (TaKaRa, Dalian, China). The following PCR program was used: one cycle at 94°C for 4 min; 32 cycles at 94°C for 1 min, 55°C for 1 min, 72°C for 1 min; and one cycle at 72°C for 7 min. PCR products were purified with a QIAquick PCR Purification Kit (BioTeke, Beijing, China), and were sequenced using an ABI 3730XL automated DNA sequencer (Applied Bio-systems, Foster City, California, U.S.A.). The sequences were deposited in GenBank (accession numbers MH023542-MH023958).

### Phylogenetic analyses and haplotype network

Sequences were aligned using MAFFT 6.864 with default parameters (Katoh et al., 2002), and were then manually adjusted using BioEdit 7.0.9 (Hall, 1999). All the sequences were assigned to different haplotypes using DnaSP 5.0 (Librado and Rozas, 2009), and these were then employed in downstream analyses. Indels were coded as presence/absence characters except for gaps caused by mononucleotide repeats, which were excluded from analyses. To evaluate genetic relationships among haplotypes, a haplotype network was constructed using NETWORK 4.6.11 with default parameters (Bandelt et al., 1999). A phylogeny of plastid haplotypes was estimated using MrBayes 3.1.2 (Yang and Rannala, 1997). Thirteen closely related taxa were selected for outgroups based on Kilian et al. (2017; Appendix 1); please note that *P. souliei* is listed under the older synonym *Melanoseris souliei*). To identify the optimal nucleotide substitution model, MrModeltest 2.3 was used (Nylander, 2004) with default parameters under the Akaike Information Criterion (AIC). For MrBayes, a random starting tree was used, and one cold and three heated chains were run simultaneously in two independent runs for 10,000,000 generations, with trees sampled every 2,000 generations. After discarding the first 25% of trees as burn-in, chain convergence was examined using TRACER 1.5.1 (http://tree.bio.ed.ac.uk/software/tracer/). In addition, to estimate genetic differentiation among *P*. *souliei* haplotypes during Pliocene to Pleistocene, molecular dating was performed under a Bayesian approach as implemented in BEAST 1.6 (Drummond and Rambaut, 2007). Analyses were run using the uncorrelated log-normal (UCLN) relaxed-clock model, and models of sequence evolution were the same as for the MrBayes analyses (GTR+I+G). The root node was constrained using a normal prior distribution [Mean: 6.9 Ma, Stdev: 0.5] by using secondary calibration based on the results of Kilian et al. (2017).

### Genetic and spatial analyses

The distribution of each plastid haplotype was plotted on a digital elevation map (DEM) derived from Shuttle Radar Topography Mission (SRTM) data (http://srtm.csi.cgiar.org/). Population diversity parameters, including number of haplotypes, haplotype diversity and nucleotide diversity were calculated from the haplotype network and phylogenetic analyses using DnaSP 5.0 (Librado and Rozas, 2009). Average within-population diversity (*H*_*S*_) and total haplotype diversity (*H*_*T*_) were calculated in PERMUT (http://www.pierroton.inra.fr/genetics/labo/Software/Permut/) to evaluate the level of genetic variation; *G*_*ST*_ and *N*_*ST*_ were used to estimate differentiation between populations. When *N*_*ST*_ is compared to *G*_*ST*_ using U-statistics, a significantly higher *N*_*ST*_ than *G*_*ST*_ usually is interpreted as indicative of phylogeographic structure. The differences between *G*_*ST*_ and *N*_*ST*_ were assessed using a permutation test with 1,000 random permutations. Samova 1.0 with default parameters (Dupanloup et al., 2002) was used to define the number of groups of populations (K) that are geographically homogenous and maximally differentiated from each other. To evaluate population genetic structure, an analysis of molecular variance (AMOVA) was used to examine the genetic variation within and between populations using Arlequin 3.1 (Excoffier et al., 2005). To investigate the correlation between genetic and geographic distance, a Mantel test was performed with 1,000 permutations in GenAlEx 6.3 (Peakall and Smouse, 2006).

### Demographic history and species distribution modeling

To evaluate whether populations experienced recent range expansion(s), Arlequin 3.1 (Excoffier et al., 2005) was used to perform pairwise mismatch distribution and neutrality analyses for the major population groups identified. Tajima's *D* and Fu's *Fs* (Tajima, 1989; Fu, 1997) were calculated to detect past demographic history using DnaSP 5.0 (Librado and Rozas, 2009), with 1000 simulated samples and a coalescent algorithm. Populations that have experienced expansion are expected to have a unimodal shape in the mismatch distribution. The sum of squared deviations (SSD) between observed and expected mismatches was also computed. Harpending's raggedness index (r) and its *P*-values were estimated to test for the significance of the population expansion model (Harpending, 1994).

Through fieldwork and using specimen records from three herbaria (PE, KUN, and HNWP; abbreviations follow Index Herbariorum: http://www.cvh.ac.cn/), a total of 159 species occurrence points were compiled for species distribution modeling (Figure S1, and these data were submitted to PANGAEA, accession number 10.1594/PANGAEA.887276). Nineteen bioclimatic variables were obtained from WorldClim 1.4, with a resolution of 1 km^2^ (Collins et al., 2004). The following three ecologically relevant bioclimatic variables were retained after eliminating highly correlated variables (i.e., *r* ≥ 0.75) (Hijmans et al., 2005): precipitation of the wettest quarter, mean temperature of the warmest quarter and precipitation seasonality (coefficient of variation). The following three layers from the Model for Interdisciplinary Research on Climate (MIROC) (Hasumi and Emori, 2004) were employed for distribution modeling: the current layer, the Mid-Holocene layer (*c*. 6 ka), and the Last Glacial Maximum (*c*. 22 ka) layer. All models were run in MaxEnt 3.2 with 10 replicates (Phillips et al., 2006), and we partitioned the locality data into training and testing data sets (75 and 25%, respectively) to evaluate the quality of the model.

## Results

### Sequence characteristics, phylogenetic relationships, and haplotype distribution

**Figure 2:**
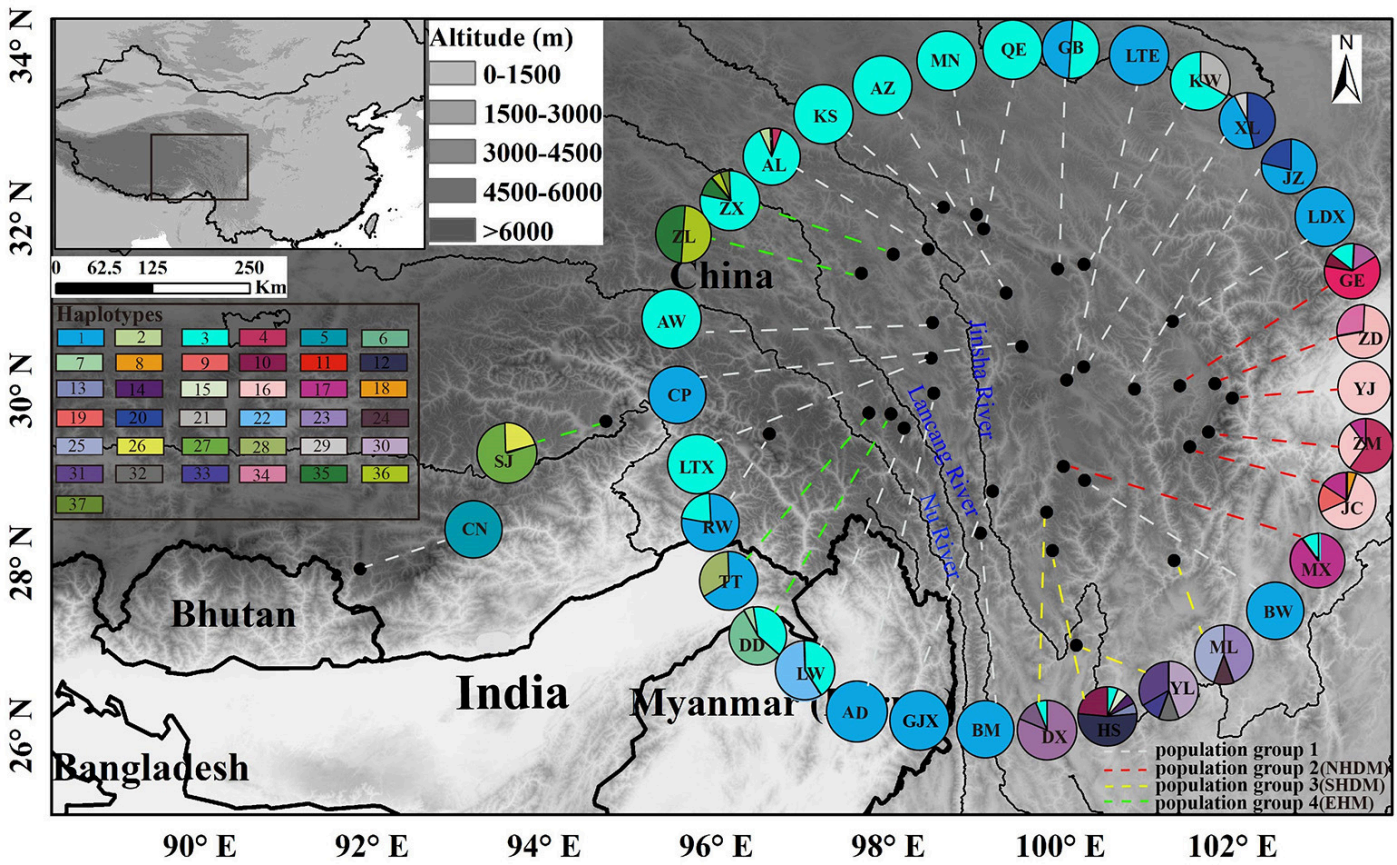
The distribution of 37 cpDNA haplotypes and the four population groups identified within 36 populations of *Parasyncalathium souliei*. Four population groups were defined according to the haplogroups. The few populations (i.e., KW, AL, GE, MX, DX, HS, DD, TT, and ZX) that contained haplotypes from multiple haplogroups were assigned to population groups based on the proportion of haplotypes from each group.

**Figure 3:**
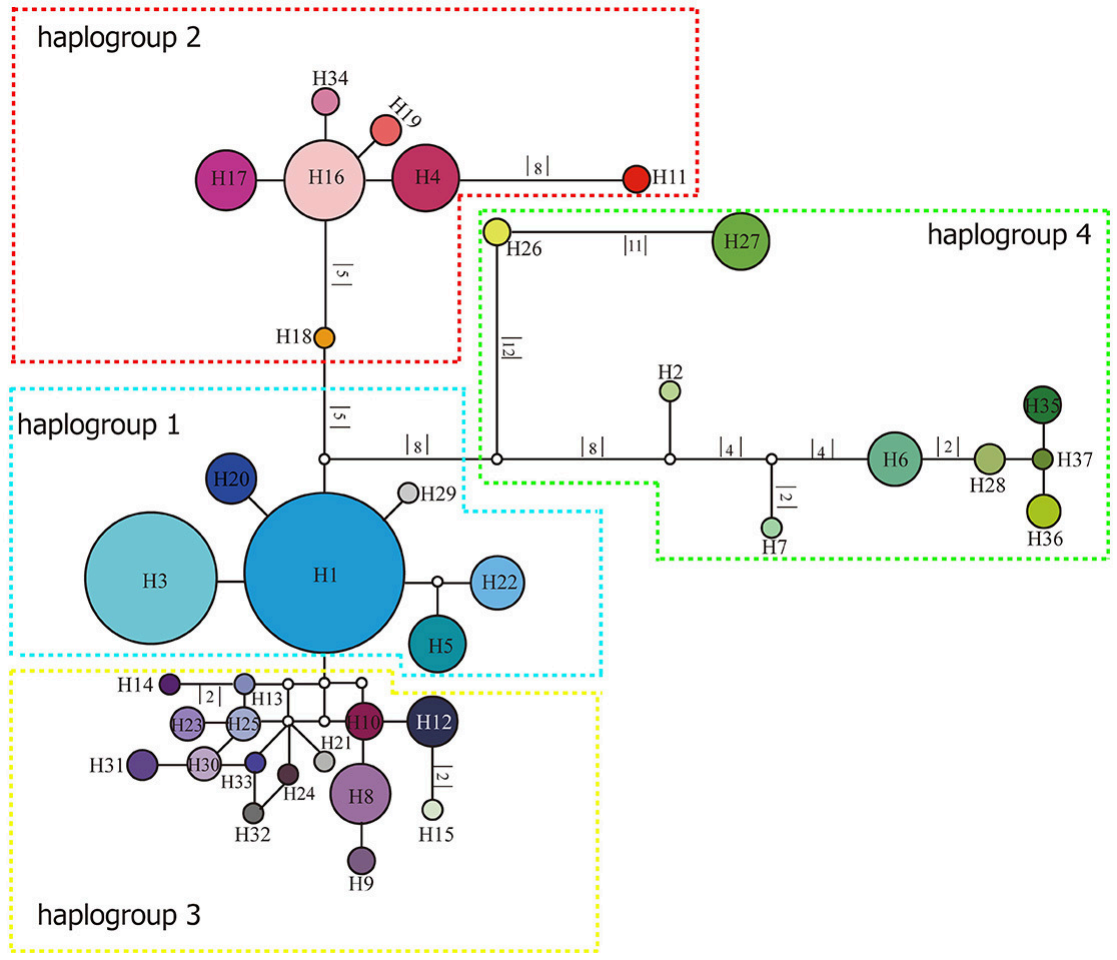
Network of relationships among the 37 cpDNA haplotypes found in *Parasyncalathium souliei*. Missing haplotypes are represented by white circles. The sizes of circles are approximately proportional to sample size. Branch lengths are indicated by the numbers along branches.

**Figure 4:**
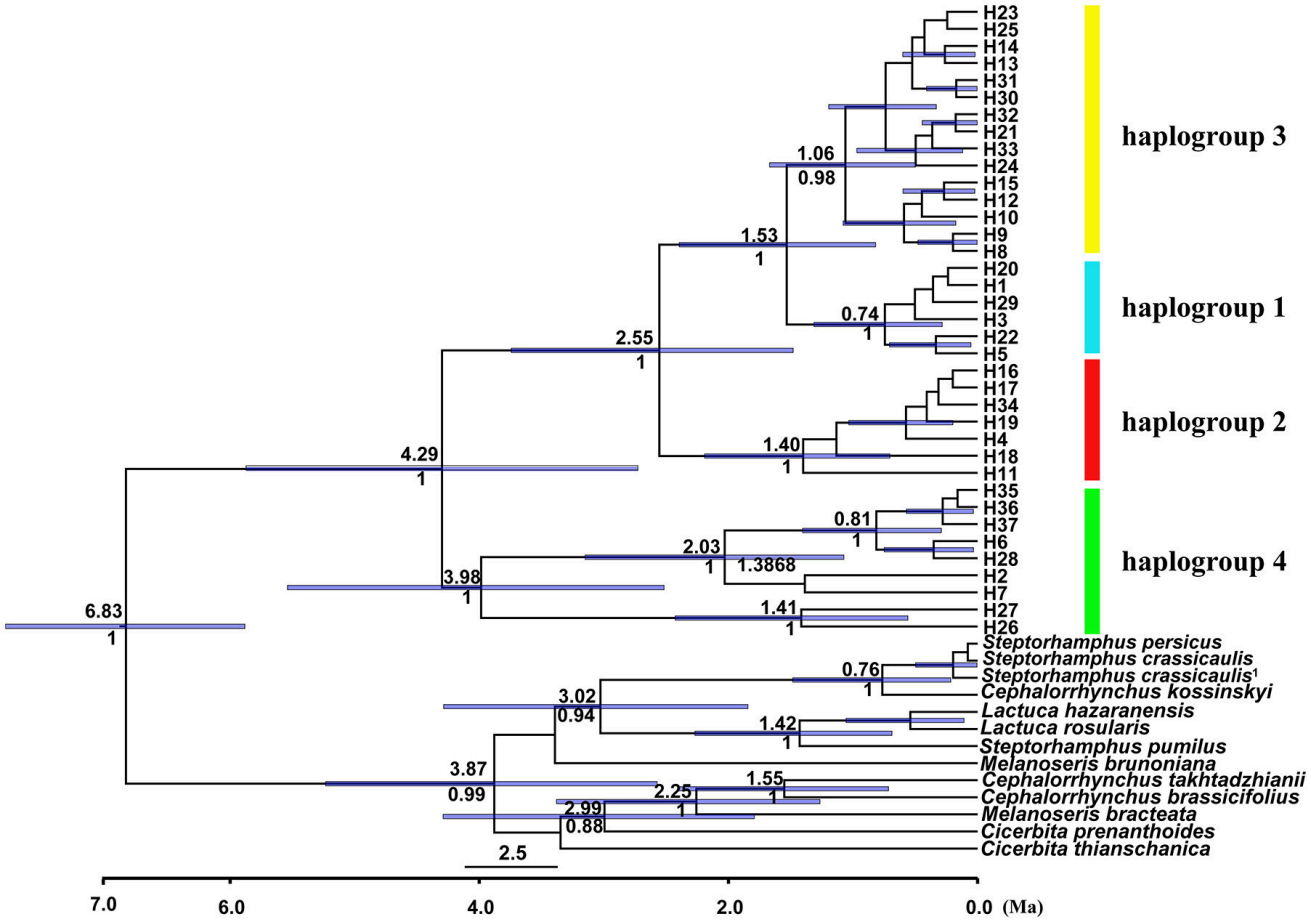
BEAST-derived chronogram for cpDNA haplotypes of *Parasyncalathium souliei*, indicating the four main haplogroups. For deeper nodes, the 95% highest posterior density interval (purple bars), the time of divergence (above branch) and posterior probabilities > 0.5 (below branch) are provided.

The length of the concatenated two-locus alignment was 1507 bp, including 77 parsimony-informative sites within *P. souliei*. A total of 86 substitutions and eight indels (not including poly-A and poly-T repeats) were detected (Appendix 2). MrModeltest identified GTR+I+G as the optimal nucleotide substitution model. A total of 37 haplotypes (H1–H37) were identified across all 417 individuals (accession number MH023959-MH023995), with 15 populations fixed for a single haplotype (Figure 2, Table 1). The most frequent and widespread haplotype (H1) was found in 141 individuals (33.9%) and 15 populations (41.7%), and the second most frequent haplotype (H3) was found in 91 individuals (21.9%) and 14 populations (38.9%). The MrBayes phylogeny of haplotypes revealed that all *P*. *souliei* haplotypes formed a well-supported clade (*PP* = 1.00). This is congruent with the result of the network and the BEAST-based chronogram. Four haplogroups were identified based on the phylogenetic and network analyses (Figures 3, 4). In haplogroup 1, the most frequent haplotype H1 was shared by approximately half of all populations (41.7%), and haplotype H3 was widely distributed, but most common in more northern regions (Figure 2). Two private haplotypes (H5 and H22) were unique to populations CN and LW, respectively. Haplotypes in haplogroup 2 (Figure 2) were restricted to six populations (GE, ZD, MX, JC, ZM, and YJ) in the NHDM, and five of them possessed more than two haplotypes (Figure 2). Haplotypes in haplogroup 3 were restricted to the SHDM (Figure 2), with all populations in this region containing three or more haplotypes. Haplotypes in haplogroup 4 were restricted to five populations (ZX, ZL, TT, DD, and SJ) in the QTP (Figure 2). These same four haplogroups were recovered as major clades in the haplotype phylogeny (Figure 4). The molecular dating analysis based on this tree recovered a Pliocene to Pleistocene diversification of these four *P. souliei* haplogroups (Figure 4).

**Table 1:** Details of sampling locations, number of individuals, geographic coordinates, elevation and haplotypes of *Parasyncalathium souliei*.

**Population abbreviation**	**Sample location**	**Sample size**	**Latitude/Longitude**	**Elevation (m)**	**Haplotype (sample numbers)**
AD	Ang-duo	9	29.96320° N/98.52820° E	4,500	H1(9)
AL	Ai-la-shan	16	31.63333° N/98.46111° E	4,682	H2(1) H3(14) H4(1)
AW	A-wang	14	31.52670° N/98.51310° E	4,075	H3(14)
AZ	An-zi	12	31.12430° N/99.36820° E	3,876	H1(12)
BM	Bai-ma-shan	13	28.34139° N/99.06944° E	4,325	H1(13)
BW	Bo-wa-shan	11	28.95249° N/100.27816° E	3,950	H1(11)
CN	Cuo-na	12	27.92364° N/91.85883° E	4,329	H5(12)
CP	Cuo-pu	5	30.50620° N/99.55162° E	4,256	H1(5)
DD	Dong da-la	18	29.72214° N/98.02914° E	4,920	H1(7) H6(10) H7(1)
DX	Da-xue-shan	16	28.58389° N/99.83833° E	4,270	H3(1) H8(13) H9(2)
GB	Gan-bai	18	31.40388° N/99.96598° E	4,430	H1(9) H3(9)
GE	Gao-er Si	13	30.04778° N/101.3858° E	4,322	H3(2) H4(8) H10(1) H11(2)
GJX	Ga-jing-xue-shan	8	28.82390° N/99.20890° E	4,370	H1(8)
HS	Hong-shan	17	28.13611° N/99.90500° E	4,480	H3(1) H10(4) H12(9) H13(1) H14(1) H15(1)
JC	Ji-chou-shan	19	29.34258° N/101.50138° E	4,426	H16(12) H17(3) H18(1) H19(3)
JZ	Jian-zi-wan-shan	14	30.01111° N/100.85778° E	4,459	H1(11) H20(3)
KS	Ke-ni	4	32.11917° N/98.64028° E	3,798	H3(4)
KW	Ka-wa	3	31.45917° N/100.27361° E	4,000	H3(2) H21(1)
LDX	Long-deng xiang	12	30.79470° N/101.29930° E	3,685	H1(12)
LTE	Li-tang	17	30.11528° N/100.07167° E	4,315	H1(17)
LTX	La-tuo-xiang	10	30.37030° N/98.49560° E	4,300	H3(10)
LW	La-wu-shan	17	29.55972° N/98.18406° E	4,310	H1(7) H22(10)
ML	Mu-li	9	28.02111° N/101.32111° E	4,027	H23(4) H24(1) H25(4)
MN	Ma-ni-gan-ge	9	32.03278° N/99.02528° E	4,275	H3(9)
MX	Ma-xiong gou	10	29.11441° N/100.03296° E	4,465	H3(1) H17(9)
QE	Que-er-shan	8	31.86764° N/99.10529° E	4,104	H3(8)
RW	Ran-wu	10	29.49222° N/96.61611° E	4,360	H1(9) H3(1)
SJ	Se-ji-la-shan	13	29.63778° N/94.71472° E	4,100	H26(2) H27(11)
TT	Tian-tuo	9	29.73611° N/97.77194° E	3,880	H1(6) H28(3)
XL	Xin-long	13	30.27000° N/100.26750° E	4,350	H1(6) H20(6) H29(1)
YJ	Ya-jia-geng	7	29.90806° N/101.99686° E	3,826	H16(7)
YL	Yu-long	9	27.03806° N/100.18139° E	4,200	H30(4) H31(3) H32(1) H33(1)
ZD	Zhe-duo-shan	7	30.07503° N/101.79605° E	4,195	H16(5) H34(2)
ZL	Zong-la-shan	6	31.35301° N /97.68640° E	4,350	H35(3) H36(3)
ZM	Zi-mei-shan	10	29.51390° N/101.72100° E	4,200	H4(6) H16(3) H17(1)
ZX	Zi-xia	19	31.57470° N/98.05530° E	4,000	H3(15) H35(2) H36(1) H37(1)

Because there was generally strong geographic localization of these haplogroups, four population groups were defined according to the haplogroups (Figure 2). The few populations that contained haplotypes from multiple haplogroups were assigned to population groups based on the proportion of haplotypes from each group. For example, populations KW and AL, GE and MX, DX and HS, and DD were assigned to population groups 1, 2, 3 and 4, respectively, based on the majority of individuals possessing haplotypes from those haplogroups. Population TT was assigned to population group 4 based on the large number of individuals with haplotypes from haplogroup 4, and population ZX was assigned to population group 4 based on the presence of 3 haplotypes from haplogroup 4.

### Genetic analyses

Total plastid genetic diversity in *P. soulei* was high (*H*_*T*_ = 0.838), while average within-population diversity was low (*H*_*S*_ = 0.290) (Table 2). Overall haplotype diversity (*Hd*) was 0.828 and nucleotide diversity (π) was 0.00685. Genetic diversity was relatively low within most populations (Appendix 3), and the highest genetic diversity was found in population GE (*Hd* = 0.769, π = 0.00987). The pairwise between-population differentiation (*F*_*ST*_) was also highly variable (Appendix 4). Haplotype diversity of the four population groups (*Hd*) ranged from 0.604 to 0.888, while nucleotide diversity (π) ranged 0.0009–0.0140 (Table 2). Genetic differentiation was remarkably high (*G*_*ST*_ = 0.653 and *N*_*ST*_ = 0.797, *P* < 0.05), which is usually interpreted as indicative of phylogeographic structure. AMOVA revealed strong population variation among populations, with the highest *F*_*ST*_ value obtained when samples were divided into four population groups (*F*_*ST*_ = 0.8102, Table 3). The AMOVA revealed that 60.85% of the genetic variation was explained by differences among the four populations groups, suggesting a significant regional population substructure, and that 20.17% was partitioned among populations within population groups. Only 18.98% was partitioned within populations, consistent with the relatively low within-population diversity (*H*_*S*_ = 0.290) (Table 3). SAMOVA failed to detect meaningful spatial population structure, as the *F*_*CT*_ values fluctuated with increasing values of K rather than showing a distinct highest value (Appendix 5). The Mantel test (*r* = 0.009; *P* = 0.13) did not find a statistically significant pattern of isolation by distance for *P. souliei*.

**Table 2:** Results of genetic and mismatch analyses in *Parasyncalathium souliei*, including number of individuals (n) and kinds of haplotypes (*K*_*h*_), haplotype diversity (*Hd*), nucleotide diversity (π), Fu's *Fs* (*Fs*), Tajima's *D* (D_T_),τ = 2ut, (where t is the expansion time and μ is the mutation rate per generation), pre-expansion population size (θ_0_), post-expansion population Size (θ_1_), sum of squared deviations (SSD, *P*-value), and Harpending's raggedness index (RAG, *P*-value).

**Population group**	***n***	***K_*h*_***	**π**	***Hd***	***F_*S*_*/*P*-value**	**D_T_/*P*-value**	**SSD/*P*-value**	**RAG/*P*-value**	**θ_0_**	**θ_1_**	**τ**
1	235	6	0.0009	0.60	−1.822/0.274	−2.302/0.000	0.0080/0.023	0.0919/0.011	0.0000	99999.00	0.881
2	66	7	0.0021	0.75	0.246/0.597	−0.61/0.044	0.0202/0.048	0.1011/0.065	0.0040	99999.00	1.193
3	51	15	0.0019	0.89	−4.852/0.026	−0.92/0.558	0.0028/0.517	0.0263/0.6550	0.0000	18.438	2.889
4	65	9	0.0140	0.86	17.214/ 0.002	2.67/0.554	0.0652/0.000	0.0723/0.000	0.0000	59.063	29.496
Total	417	37	0.0069	0.83	−0.342/0.566	−0.643/0.288	0.7432/0.000	0.0316/1.000	0.0000	6.0310	29.729

**Table 3:** Structure of genetic variation within and among four population groups of *Parasyncalathium souliei* (*F*_*CT*_, differentiation among groups within the species; *F*_*SC*_, differentiation among populations within groups; *F*_*ST*_, differentiation among populations within the species).

**Source of variation**	**Percentage of variation**	***F*-statistic**
**ACROSS ALL POPULATION GROUPS**		
Among groups	60.85	
Among populations within groups	20.17	*F_*CT*_* = 0.60848
Within populations	18.98	*F_*SC*_* = 0.51525
Total		*F_*ST*_* = 0.81021
**GROUP 1 VS. GROUP 2**		
Among groups	86.06	
Among populations within groups	5.99	*F_*CT*_* = 0.86068^***^
Within populations	7.95	*F_*SC*_* = 0.42960^***^
Total		*F_*ST*_* = 0.92053^***^
**GROUP 1 VS. GROUP 3**		
Among groups	70.46	
Among populations within groups	16.69	*F_*CT*_* = 0.70460^***^
Within populations	12.85	*F_*SC*_* = 0.56508^***^
Total		*F_*ST*_* = 0.87152^***^
**GROUP 1 VS. GROUP 4**		
Among groups	53.04	
Among populations within groups	25.36	*F_*CT*_* = 0.53040^***^
Within populations	21.60	*F_*SC*_* = 0.53998^***^
Total		*F_*ST*_* = 0.78398^***^
**GROUP 2 VS. GROUP 3**		
Among groups	76.94	
Among populations within groups	8.34	*F_*CT*_* = 0.76943^***^
Within populations	14.72	*F_*SC*_* = 0.36152^***^
Total		*F_*ST*_* = 0.85279^***^
**GROUP 2 VS. GROUP 4**		
Among groups	46.84	
Among populations within groups	27.87	*F_*CT*_* = 0.46838^***^
Within populations	25.29	*F_*SC*_* = 0.52422^***^
Total		*F_*ST*_* = 0.74707^***^
**GROUP 3 VS. GROUP 4**		
Among groups	30.90	
Among populations within groups	39.98	*F_*CT*_* = 0.30903^*^
Within populations	29.12	*F_*SC*_* = 0.57856^***^
Total		*F_*ST*_* = 0.70880^***^

*** P < 0.0001;

* *P < 0.005*.

### Estimates of historical demography and species distribution modeling

**Figure 5:**
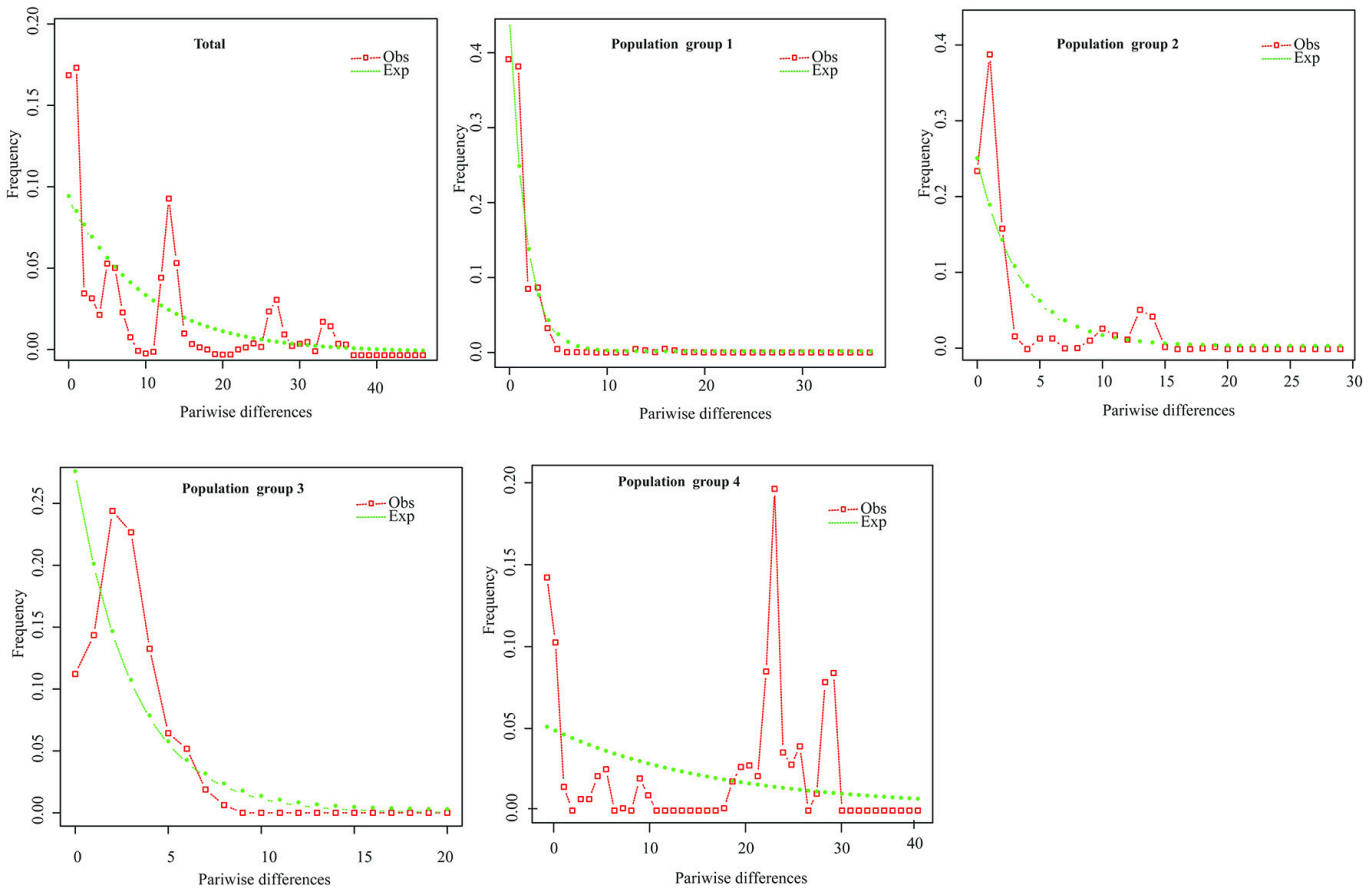
Mismatch distribution analysis plots across all populations and for the four population groups of *P. souliei*.

Hierarchical mismatch analyses indicated that distributions of pairwise differences for population groups 2, 3, 4, and the entire species were ragged or multimodal (Figure 5), which rejected the hypothesis of demographic population expansion of these population groups. The SSD and RAG index (*P* < 0.05) also rejected the historical population expansion of population group 1 despite its unimodal pattern in the mismatch analysis (Table 3,Figure 5).

Species distribution modeling yielded an over-prediction of the geographic distribution of *P. souliei* to the north and west of its known range, albeit with weak confidence in these areas (Figure S2). Overall, the predicted ranges of *P. souliei* in the Mid-Holocene and Last Glacial Maximum were very similar to that of the predicted current distribution; the predicted range was slightly smaller during the Mid-Holocene and was shifted slightly northward during the Last Glacial Maximum but still with the high climatic suitability of HDM (Figure S2).

## Discussion

### Phylogeography

Compared to the genetic diversity of 170 plant species compiled by Petit et al. (2005), the plastid haplotype diversity of *P. souliei* (*H*_*T*_ = 0.838) is higher than average (*H*_*T*_ = 0.670). High plastid diversity has also been detected in other QTP-HDM species such as *Eriophyton wallichii* Benth. (Lamiaceae): *H*_T_ = 0.983; *Thalictrum squamiferum* Lecoy. (Ranunculaceae): *H*_T_ = 0.973; *Paraquilegia microphylla* (Royle) J. R. Drumm and Hutch. (Ranunculaceae): *H*_T_ = 0.984; and *Chionocharis hookeri* (C. B. Clarke) I. M. Johnst. (Boraginaceae): *H*_T_ = 0.935 (Luo et al., 2016). Chloroplast genetic differentiation among populations is relatively high (*N*_*ST*_ = 0.797; *G*_*ST*_ = 0.653, *P* < 0.05), which may result from the topographic complexity of the QTP-HDM. Considering the lack of spatial population genetic structure discernable from Samova suggesting such high inter-population differentiation may have been driven by genetic isolation and drift arising from dispersal limitation and topographic effects during Pleistocene full-glacial periods (Xing and Ree, 2017).

Our analyses imply that *P. souliei* diversified in the Pliocene to Pleistocene and that the current geographic range of the species may result from a complex pattern of long-term population stability. Pliocene to Pleistocene diversification has also been inferred in other QTP-HDM taxa such as *Solms-laubachia* Muschl. (Brassicaceae) (Yue et al., 2009), *Baimashania* Al-Shehbaz (Brassicaceae) (Koch et al., 2012), and *Meconopsis* Vig. (Papaveraceae) (Wen et al., 2014), suggesting that this period was an important period for species/lineage differentiation in the QTP-HDM. These dates coincide with later estimates for the age of the uplift of the QTP (Li and Fang, 1999) and the formation of the Hengduan Mountains (Shi et al., 1998; Akciz et al., 2008). The highest levels of within-population variation in *P. souliei* occur in the HDM, and particularly the SHDM, and this combined with the many unique haplotypes found there suggest that *P. souliei* populations may have been relatively stable through time in the HDM. The HDM is a region of highly heterogeneous microclimates and it has been suggested to be a refugium for many plant species during Pleistocene glacial periods, such as *Taxus wallichiana* Zucc. (Taxaceae) (Liu et al., 2013) and *Quercus aquifolioides* Rehder and E. H. Wilson (Fagaceae) (Du et al., 2017). Our species distribution modeling analyses (Figure S2) also suggest that climatic conditions favorable for the persistence of *P. souliei* existed across all modeled periods. In contrast with previous studies evaluating possible glacial effects on phylogeographic patterns in the QTP-HDM region (e.g., Zhang et al., 2005; Yang et al., 2008), our distribution modeling analyses indicate relative stasis of *P. souliei* populations during the later Pleistocene, rather than clear contraction (Figure S2). This relatively unusual demographic scenario may be explained by the wide ecological tolerance of *P. souliei* combined with the extreme topoclimatic complexity of the QTP-HDM region, which may have provided suitable areas for the *in situ* persistence of *P. souliei* even during glaciations. This contrasts to the general “contraction–expansion” scenario usually described in other plants (e.g., Yan et al., 2012). Away from the HDM, unique haplotypes are also found in other parts of the range of *P. souliei*, including the QTP, although within-population diversity is generally lower in these areas. It is important to note that the deepest phylogenetic split among haplotypes is between haplogroup 4 (group EHM, with haplotypes in the western part of the species range) and all other haplogroups (which are centered in the eastern part of the range). This implies that populations could have survived in other regions of the range away from the HDM as well, suggesting vicariance of at least some populations. However, the widespread nature of the relatively young haplotypes 1 and 3 (Figure 2) in QTP populations may reflect more recently established populations in this area.

### Incorporating haplotype information in phylogeographic regionalization

Biogeographical boundaries delineate the basic macrounits of diversity in biogeography, conservation, and macroecology (Daru et al., 2016, 2017). Species composition in different biogeographic areas reflects the historical processes, such as orogeny and glaciations, that have helped to shape the present-day distribution of biodiversity (Mackey et al., 2007; Kreft and Jetz, 2010). Traditionally, biogeographical regions have been defined via qualitative and/or quantitative approaches that use geographical distribution and landscape features to delimit hierarchical floristic regions (Takhtajan, 1986; Wu and Wu, 1996; González-Orozco et al., 2014; Zhang et al., 2016). Recently, phylogenetic information has begun to be incorporated into regionalization schemes by considering phylogenetic dispersal, extinction, speciation, and niche conservatism to define assembly of species into distinct biogeographic units (Daru et al., 2017). Within the QTP-HDM hotspot region, two dominant methods have been used to delimit biogeographic regions: (1) based on the presence and absence data at the family, genus and species level (Wu and Wu, 1996), and (2) based on patterns of similarity in taxonomic composition (Zhang et al., 2016). We propose that the history of individual species, and in particular those that are widely distributed within biodiversity hotspots, may provide key information to help delineate phylogeoregions, such as in the QTP-HDM (Appendix 6). Such phylogeoregions have the advantage of incorporating information at relatively shallow (i.e., recent) time scales, and may or may not correspond to existing proposed biogeographic regions. For example, Amarilla et al. (2015) identified three clades of haplotypes within the grass *Munroa argentina* Griseb. that correspond to previously identified biogeographic regions in the South American Transition Zone (SATZ).

As with the *Munroa* example, our phylogeographic results in *Parasyncalathium souliei* are largely consistent with the biogeographic regions proposed by Wu and Wu (1996). Three of the four haplogroups detected in *P. souliei* (groups 2–4; Figure 2) are geographically localized within previously proposed subregions: haplogroup 2 was distributed through the NHDM; haplogroup 3 was located through the SHDM; and haplogroup 4 was mainly distributed in the eastern Himalayan region (hereafter called EHM), including the Three river-gorges and Southeast Xizang subregions (Figure 2). In contrast, haplotypes from haplogroup 1 are more widely distributed, ranging from the Paleotropical floristic kingdom (population CN in Figure 2) to the HDM (Wu and Wu, 1996), although most of the individual haplotypes in haplogroup 1 are highly localized (Figure 2). The wide distribution of sister haplotypes 1 and 3 is difficult to accommodate in hypotheses of phylogeographic regionalization (Figure 2). Most of the populations that are fixed for haplotypes in haplogroup 1 are at higher elevations and/or are further north, and it is possible that these populations have been established more recently by one or a few ancestral population(s) dominated by haplotypes in this haplogroup. However, it is difficult to pinpoint the cause for this pattern given available evidence.

Based on our results and a survey of the literature (e.g., Zhang et al., 2016), we propose that the distribution of private haplotypes in *P. souliei* may reflect a putative set of phylogeoregions that roughly follow the biogeographic regions of Wu and Wu (1996), with some important differences. Overall, we suggest that the QTP-HDM region is best divided into three phylogeoregions (I, II, and III; Figure 1B) rather than the more complicated subdivisions proposed by Wu and Wu (1996). More specifically, there are three main differences between the earlier qualitative division of Wu and Wu (1996; Figure 1A), which is based on the geographical distribution of endemic species, genera, and families, and our proposed phylogeographic divisions (Figure 1B). First, we find no support (i.e., private haplotypes; Figure 2) in *P. souliei* for two of the subkingdoms (Figure 1A; III: East Asia floristic kingdom; IV: Paleotropical floristic kingdom) of Wu and Wu (1996). Likewise, other phylogeographic studies that have involved this region (region IVG 24 in Figure 1A) have not found private haplotypes that distinguish these two regions (e.g., Li et al., 2011; Jia et al., 2012; Liu et al., 2013). It is therefore more reasonable to assign IVG 24 of Wu and Wu (1996) to the East Asia floristic kingdom (Figure 1).

Secondly, our results suggest that the Jinsha River may mark an important phylogeographic boundary between what we propose is a new Himalayan phylogeoregion (region III; Figure 1B) and the North and South HDM phylogeoregions (regions I and II; Figure 1B). The areas east and west of the Jinsha River are profoundly different in terms of ecology and species diversity and these differences are associated with the uplift of the QTP and the north/south barrier to the monsoon climate created by the Gaoligong and Nushan Mountains (Liu et al., 2013). This differentiation between the EHM and HDM regions is also supported by paleoclimatic evidence (Kou et al., 2006). Similarly, Zhang et al. (2016) have also assigned the Three river-gorges subregion of Wu and Wu (1996) to our proposed Eastern Himalayan phylogeoregion based on quantificationally hierarchical clustering of species endemism. Likewise, a recent phylogeographic study of *Marmoritis complanata* (Dunn) A. L. Budantzev (Lamiaceae) detected what the authors called the “Ward Line-Mekong-Salween Divide” (Luo et al., 2017) as an important floristic boundary, which is consistent with our placement of the old Three river-gorges area in the EHM phylogeoregion.

Thirdly, we suggest that the marked division in the geographic distribution of haplotypes between haplogroups 2 and 3 in *P. souliei* at approximately 29° N latitude (Figure 2) may reflect another important phylogeographic boundary in the QTP-HDM, specifically the division between the North and South HDM. Hence, we tentatively suggest that the boundary of the putative NHDM and SHDM phylogeoregions be placed at this latitude (red horizontal line in Figure 1B). Significant differences in climate and soil exist north and south of this line: the NHDM is characterized of by a drier, subfrigid plateau climate dominated by alpine meadows, whereas the SHDM is characterized by a wetter, subtropical montane climate dominated by broadleaf evergreen forest (Yu et al., 1989; Li et al., 2010). The two putative phylogeoregions have also been shown to be distinctly different in soil microbiota, especially in the number of radiobacteria (Shao et al., 2005).

## Conclusions

Compared to qualitative methods of biogeographic regionalization based on the distribution of endemic plant species and quantitative methods that employ hierarchical clustering and ordination of plant communities, such as those of Wu and Wu (1996) and Zhang et al. (2016), our approach helps to identify potential barriers to dispersal and survival within individual taxa. However, we emphasize that our study cannot fully resolve the phylogeographic patterns in the QTP-HDM because it is based on a single species with a short life span. While such species may provide valuable insight into phylogeographic patterns because they have elevated mutation rates, a more complete picture of phylogeographic regions will require studies of many more taxa including a variety of growth forms in many different plant families. Such future studies will help bring about a fuller view of the biogeographic complexities of the QTP-HDM, and hence further improve our classification of biogeographic regions.

## Author contributions

HS, JZ, and HW conceived and designed this study; NL, TD, and YS analyzed the data, and MM, NL, TD, and YS wrote the manuscript; XH, WS, and DL aided in field collections. All authors read and approved the final manuscript.

### Conflict of interest statement

The authors declare that the research was conducted in the absence of any commercial or financial relationships that could be construed as a potential conflict of interest.

## Supplementary material

Figure S1 The 159 species occurrence points (black dots in figure) were for species distribution modeling.

Figure S2 Climate-based species distribution models of *P. souliei* for three geological time periods: present day, Mid-Holocene (*c*. 6 ka), and Last Glacial Maximum (*c*. 22 ka).

Appendix 1 List of sampling localities, herbarium voucher specimens, and GenBank accession numbers. Herbarium acronyms is as follows: KUN = Herbarium of Kunming Institute of Botany, Chinese Academy of Sciences.

Appendix 2 Chloroplast DNA sequence polymorphism detected at mutation sites at haplotypes in *P. souliei*.

Appendix 3 Genetic diversity within populations and correlations test. Genetic diversity within populations. (Hd indicates haplotype diversity; π indicates nucleotide diversity).

Appendix 4 Pairwise FST (blow diagonal) and *P*-values (above diagonal) among populations of *P. souliei*.

Appendix 5 Table and Figure of relationship between the fixation index (FCT; *y*-axis) and number of clusters (K; *x*-axis) in the SAMOVA of *P. souliei*.

Appendix 6 Several cases of biogeographic regionalization based on one species from Qinghai-Tibet Plateau (QTP) - Hengduan Mountains (HDM) region.
