# Supplementary figures and images for: Phylogeography of Parasyncalathium souliei (Asteraceae) and Its Potential Application in Delimiting Phylogeoregions in the Qinghai-Tibet Plateau (QTP)-Hengduan Mountains (HDM) Hotspot
Source: Front Genet. 2018 May 17;9:171. doi: 10.3389/fgene.2018.00171 (PMC5966570; doi:10.3389/fgene.2018.00171)

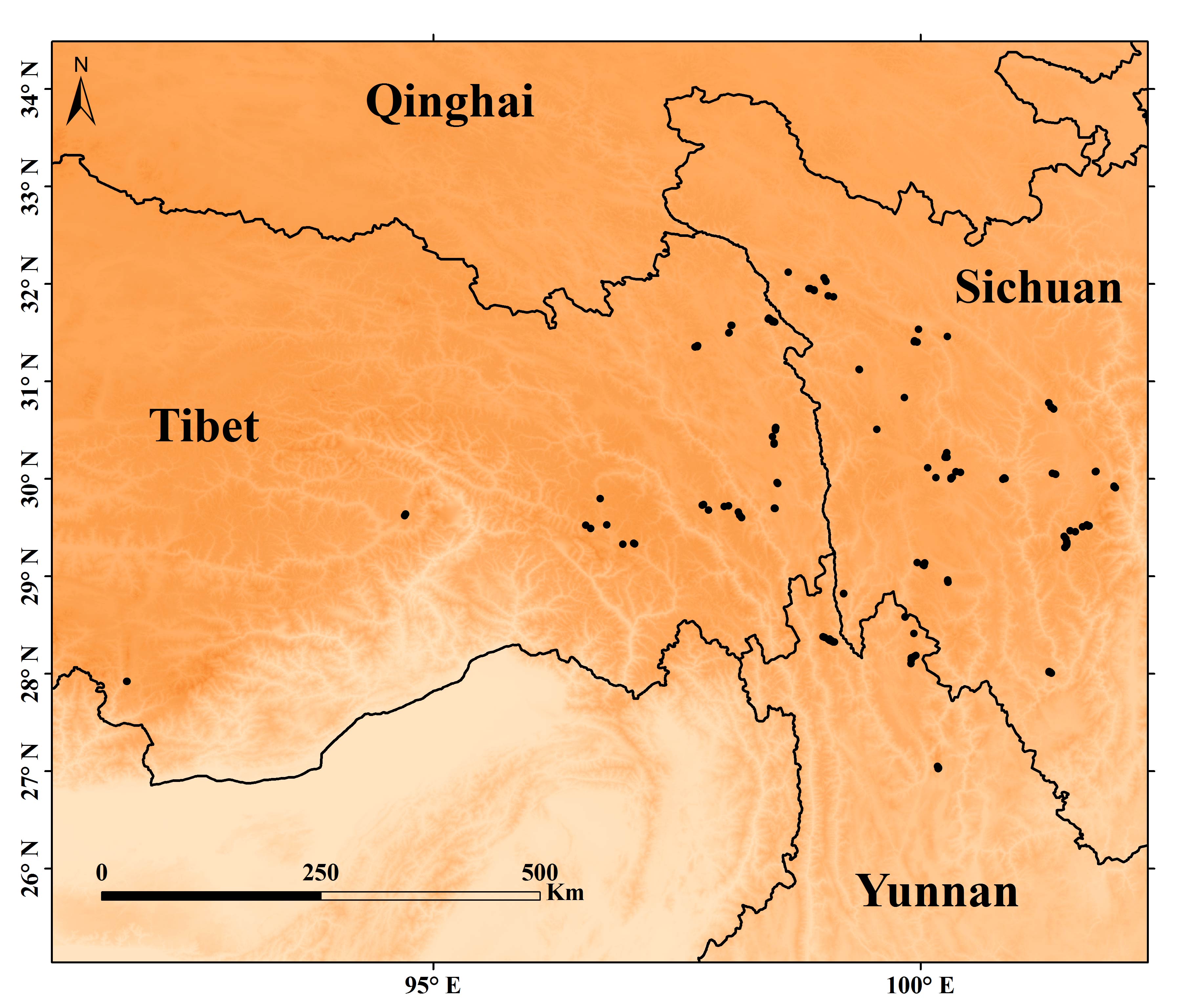

Supplement: Figure S1 — The 159 species occurrence points (black dots in figure) were for species distribution modeling. [file Image_1.JPEG]

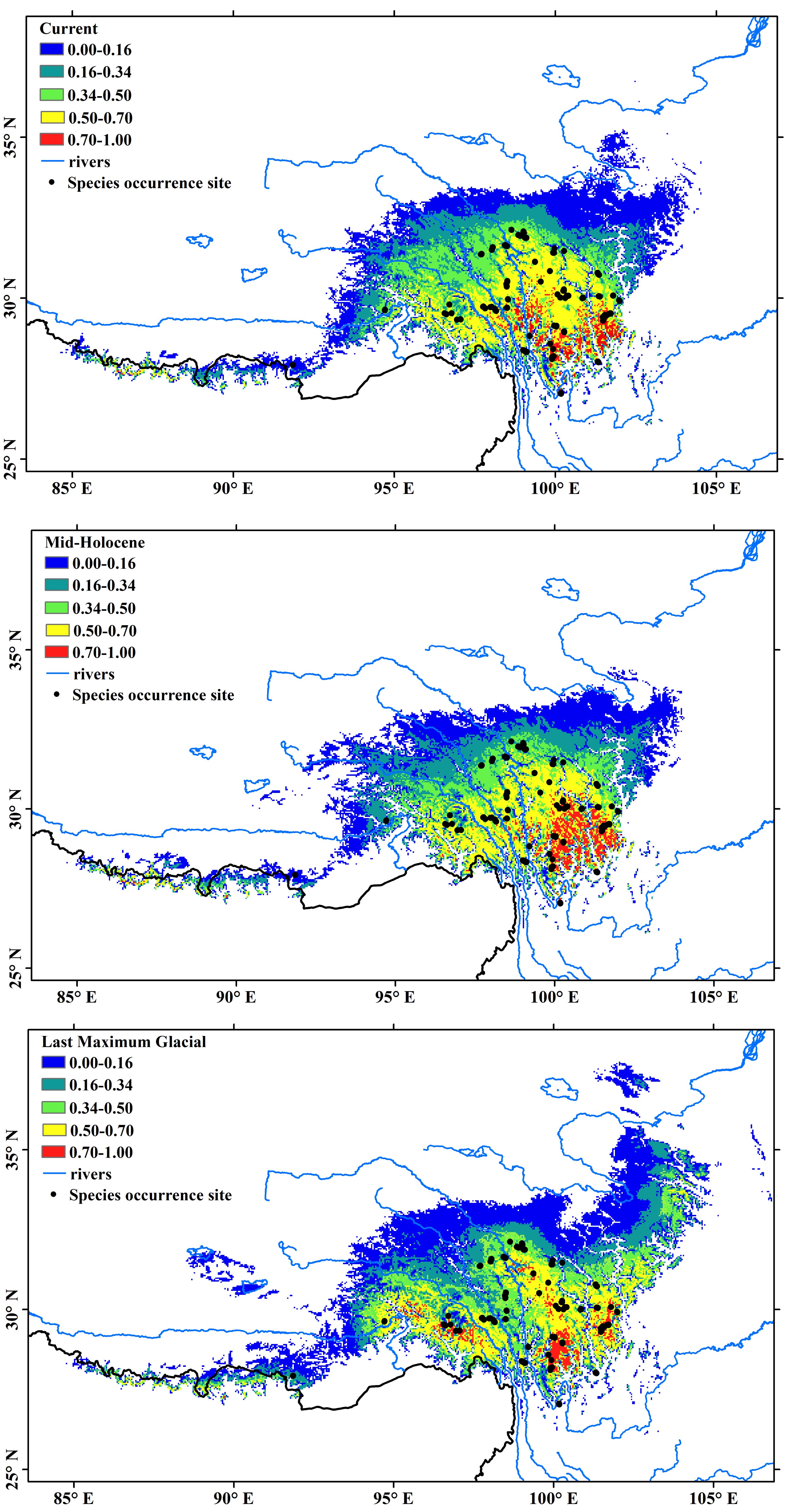

Supplement: Figure S2 — Climate-based species distribution models of P. souliei for three geological time periods: present day, Mid-Holocene (c. 6 ka), and Last Glacial Maximum (c. 22 ka). [file Image_2.JPEG]
