# Supplementary material for: Phylogeography of Parasyncalathium souliei (Asteraceae) and Its Potential Application in Delimiting Phylogeoregions in the Qinghai-Tibet Plateau (QTP)-Hengduan Mountains (HDM) Hotspot
Source: Front Genet. 2018 May 17;9:171. doi: 10.3389/fgene.2018.00171 (PMC5966570; doi:10.3389/fgene.2018.00171)
Supplement: Appendix 1 — List of sampling localities, herbarium voucher specimens, and GenBank accession numbers. Herbarium acronyms is as follows: KUN = Herbarium of Kunming Institute of Botany, Chinese Academy of Sciences. [file Table_1.docx]

Supplementary Material

**Phylogeography of *Parasyncalathium souliei* (Asteraceae) and** **its potential application in delimiting phylogeoregions in the Qinghai-Tibet Plateau (QTP) - Hengduan Mountains (HDM) hotspot**

**Nan Lin^1,2,3#^, Tao Deng^3#^, Michael J. Moore^4^, Yanxia Sun^1^, Xianhan Huang^3^, Wenguang Sun^3^, Dong Luo^3^, Hengchang Wang^1,*^, Jianwen Zhang^3,*^, Hang Sun^3,*^**

^1^Key Laboratory of Plant Germplasm Enhancement and Specialty Agriculture, Wuhan Botanical Garden, Chinese Academy of Sciences, Wuhan, Hubei, China

^2^University of Chinese Academy of Sciences, Beijing, China

^3^Key Laboratory for Plant Diversity and Biogeography of East Asia, Kunming Institute of Botany, Chinese Academy of Sciences, Kunming, Yunnan, China;

^4^Department of Biology, Oberlin College, Oberlin, Ohio, USA;

^#^ These authors have contributed equally to this work.

**^*^ Correspondence:**

Hang Sun, [sunhang@mail.kib.ac.cn](mailto:sunhang@mail.kib.ac.cn);

Jianwen Zhang, [zhangjianwen@mail.kib.ac.cn](mailto:zhangjianwen@mail.kib.ac.cn);

Hengchang Wang, [hcwang@wbgcas.cn](mailto:hcwang@wbgcas.cn)

Appendix 1. List of sampling localities, herbarium voucher specimens, and GenBank accession numbers. Herbarium acronyms is as follows: KUN = Herbarium of Kunming Institute of Botany, Chinese Academy of Sciences.

| Population abbreviation／Taxon | Latitude/Longitude |  | GenBank accession numbers | Herbarium voucher specimen (KUN)/Reference |
| --- | --- | --- | --- | --- |
| AD | 29.96320° N/98.52820° E |  | MH023542-MH023545 | Zhang, Zhang & Gong 219 |
| AL | 31.63333° N /98.46111° E |  | MH023546-MH023561 | Zhang, Yang & Chen 798 |
| AW | 31.52670° N /98.51310° E |  | MH023562-MH023575 | Zhang, Zhang & Gong 215 |
| AZ | 31.12430° N /99.36820° E |  | MH023576-MH023588 | Zhang, Zhang & Gong 180 |
| BM | 28.34139° N /99.06944° E |  | MH023589-MH023601 | Zhang, Sun & Qian 005 |
| BW | 28.95249° N /100.27816° E |  | MH023602-MH023612 | Zhang, Zhang & Gong 264 |
| CN | 27.92364° N /91.85883° E |  | MH023613-MH023624 | ZJW-3997 |
| CP | 30.50620° N /99.55162° E |  | MH023625-MH023629 | SunH-07ZX-3659 |
| DD | 29.72214° N /98.02914° E |  | MH023630-MH023647 | Zhang, Sun & Qian 037 |
| DX | 31.40388° N /99.96598° E |  | MH023660-MH023675 | Zhang, Yang & Chen 930 |
| GB | 30.04778° N /101.3858° E |  | MH023676-MH023693 | SunH-07ZX-3479 |
| GE | 28.82390° N /99.20890° E |  | MH023694-MH023706 | Zhang 074 |
| GJX | 28.13611° N /99.90500° E |  | MH023707-MH023714 | Zhang, Zhang & Gong 244 |
| HS | 29.34258° N /101.50138° E |  | MH023715-MH023731 | SunH-07ZX-2376 |
| JC | 30.01111° N /100.85778° E |  | MH023732-MH023750 | Zhang, Zhang & Gong 035 |
| JZ | 32.11917° N /98.64028° E |  | MH023751-MH023764 | Zhang, Zhang & Gong 122 |
| KS | 31.45917° N /100.27361° E |  | MH023765-MH023768 | SunH-07ZX-3729 |
| KW | 30.79470° N /101.29930° E |  | MH023769-MH023771 | Zhang1026 |
| LDX | 28.58389° N /99.83833° E |  | MH023648-MH023659 | Zhang, Zhang & Gong 123 |
| LTE | 30.11528° N /100.07167° E |  | MH023772-MH023788 | SunH-07ZX-3366 |
| LTX | 30.37030° N /98.49560° E |  | MH023789-MH023798 | Zhang, Zhang & Gong 216 |
| LW | 29.55972° N /98.18406° E |  | MH023799-MH023815 | Zhang, Zhang & Gong 234 |
| ML | 28.02111° N /101.32111° E |  | MH023816-MH023824 | Kilian et al. 10971 (= ERS 5431) |
| MN | 32.03278° N /99.02528° E |  | MH023825-MH023833 | SunH-07ZX-3734 |
| MX | 29.11441° N /100.03296° E |  | MH023834-MH023843 | Zhang, Yang & Chen 887 |
| QE | 31.86764° N /99.10529° E |  | MH023844-MH023851 | Zhang, Yang & Chen 805A |
| RW | 29.49222° N /96.61611° E |  | MH023852-MH023861 | SunH-07ZX-2410 |
| SJ | 29.63778° N /94.71472° E |  | MH023862-MH023874 | SunH-07ZX-2821 |
| TT | 29.73611° N /97.77194° E |  | MH023875-MH023883 | SunH-07ZX-2408 |
| XL | 30.27000° N /100.26750° E |  | MH023884-MH023896 | Zhang, Yang & Chen 845 |
| YJ | 29.90806° N /101.99686° E |  | MH023897-MH023903 | Zhang, Yang & Chen 074 |
| YL | 27.03806° N /100.18139° E |  | MH023904-MH023916 | Kilian et al. 11242 (= ERS 5702) |
| ZD | 30.07503° N /101.79605° E |  | MH023917-MH023923 | Zhang, Yang & Chen 103 |
| ZL | 31.35301° N /97.68640° E |  | MH023924-MH023929 | ZJW-3205 |
| ZM | 29.51390° N /101.72100° E |  | MH023930-MH023939 | Zhou & Li 028 |
| ZX | 31.57470° N /98.05530° E |  | MH023940-MH023958 | Zhang, Zhang & Gong 213 |
| *Cephalorrhynchus brassicifolius* [(Boiss.) Tuisl](http://www.theplantlist.org/tpl/record/gcc-10691) |  |  | LT722339, LT722102 | Kilian et al. (2017) |
| *Cephalorrhynchus kossinskyi* (Krasch.) Kirp |  |  | LT722345, LT722108 | Kilian et al. (2017) |
| *Cephalorrhynchus takhtadzhianii* Sosn. |  |  | LT722352, LT722115 | Kilian et al. (2017) |
| *Cicerbita prenanthoides* [(M. Bieb.) Beauverd](http://www.theplantlist.org/tpl/record/gcc-115928) |  |  | LT722355, LT722118 | Kilian et al. (2017) |
| *Cicerbita thianschanica* [(Regel & Schmalh.) Beauverd](http://www.theplantlist.org/tpl/record/gcc-83607) |  |  | LT722356, LT722119 | Kilian et al. (2017) |
| *Lactuca hazaranensis* Djavadi & N. Kilian |  |  | LT722359, LT722122 | Kilian et al. (2017) |
| *Lactuca rosularis* [Boiss.](http://www.theplantlist.org/tpl/record/gcc-109457) |  |  | LT722364, LT722127 | Kilian et al. (2017) |
| *Melanoseris bracteate*  [Hook.f. & Thomson ex Hook.f.](http://www.theplantlist.org/tpl1.1/record/gcc-121737) |  |  | LT722367, LT722131 | Kilian et al. (2017) |
| *Melanoseris brunoniana* (Wall. ex DC.) N. Kilian & Ze H. Wang |  |  | LT722368, LT722132 | Kilian et al. (2017) |
| *Steptorhamphus crassicaulis* [(Trautv.) Kirp.](http://www.theplantlist.org/tpl1.1/record/gcc-34566) |  |  | LT722372, LT722140 | Kilian et al. (2017) |
| *Steptorhamphus crassicaulis* [(Trautv.) Kirp.](http://www.theplantlist.org/tpl1.1/record/gcc-34566) 1 |  |  | LT722371, LT722141 | Kilian et al. (2017) |
| *Steptorhamphus persicus* [Grossh.](http://www.theplantlist.org/tpl1.1/record/gcc-44125) |  |  | LT722374, LT722143 | Kilian et al. (2017) |
| *Steptorhamphus pumilus* [(Rech.f. & Tuisl) Tuisl](http://www.theplantlist.org/tpl1.1/record/gcc-41391) |  |  | LT722375, LT722144 | Kilian et al. (2017) |
