# Supplementary material for: Phylogeography of Parasyncalathium souliei (Asteraceae) and Its Potential Application in Delimiting Phylogeoregions in the Qinghai-Tibet Plateau (QTP)-Hengduan Mountains (HDM) Hotspot
Source: Front Genet. 2018 May 17;9:171. doi: 10.3389/fgene.2018.00171 (PMC5966570; doi:10.3389/fgene.2018.00171)
Supplement: Appendix 2 — Chloroplast DNA sequence polymorphism detected at mutation sites at haplotypes in P. souliei. [file Table_2.docx]

Supplementary Material

**Phylogeography of *Parasyncalathium souliei* (Asteraceae) and** **its potential application in delimiting phylogeoregions in the Qinghai-Tibet Plateau (QTP) - Hengduan Mountains (HDM) hotspot**

**Nan Lin^1,2,3#^, Tao Deng^3#^, Michael J. Moore^4^, Yanxia Sun^1^, Xianhan Huang^3^, Wenguang Sun^3^, Dong Luo^3^, Hengchang Wang^1,*^, Jianwen Zhang^3,*^, Hang Sun^3,*^**

^1^Key Laboratory of Plant Germplasm Enhancement and Specialty Agriculture, Wuhan Botanical Garden, Chinese Academy of Sciences, Wuhan, Hubei, China

^2^University of Chinese Academy of Sciences, Beijing, China

^3^Key Laboratory for Plant Diversity and Biogeography of East Asia, Kunming Institute of Botany, Chinese Academy of Sciences, Kunming, Yunnan, China;

^4^Department of Biology, Oberlin College, Oberlin, Ohio, USA;

^#^ These authors have contributed equally to this work.

**^*^ Correspondence:**

Hang Sun, [sunhang@mail.kib.ac.cn](mailto:sunhang@mail.kib.ac.cn);

Jianwen Zhang, [zhangjianwen@mail.kib.ac.cn](mailto:zhangjianwen@mail.kib.ac.cn);

Hengchang Wang, [hcwang@wbgcas.cn](mailto:hcwang@wbgcas.cn)

Appendix 2. Chloroplast DNA sequence polymorphism detected at mutation sites at haplotypes in *P. souliei.*

|  | H1 | H2 | H3 | H4 | H5 | H6 | H7 | H8 | H9 | H10 | H11 | H12 | H13 | H14 | H15 | H16 | H17 | H18 | H19 | H20 | H21 | H22 | H23 | H24 | H25 | H26 | H27 | H28 | H29 | H30 | H31 | H32 | H33 | H34 | H35 | H36 | H37 |
| --- | --- | --- | --- | --- | --- | --- | --- | --- | --- | --- | --- | --- | --- | --- | --- | --- | --- | --- | --- | --- | --- | --- | --- | --- | --- | --- | --- | --- | --- | --- | --- | --- | --- | --- | --- | --- | --- |
| 6 | C | C | C | C | C | C | C | C | C | C | C | C | C | C | C | C | C | C | C | C | C | C | C | C | C | C | C | C | C | C | C | C | C | T | C | C | C |
| 33 | G | G | G | G | G | G | G | G | G | G | G | G | G | G | G | G | G | G | G | G | G | G | G | G | G | G | G | G | G | A | A | A | A | G | G | G | G |
| 104 | A | A | A | G | A | G | G | A | A | A | G | A | A | A | A | G | G | A | G | A | A | A | A | A | A | A | G | G | A | A | A | A | A | G | G | G | G |
| 134 | - | - | - | - | - | - | - | - | - | - | - | - | - | - | - | - | - | - | - | - | - | - | - | - | - | - | - | a | - | - | - | - | - | - | - | - | - |
| 162 | C | C | C | C | C | C | C | C | C | C | C | C | C | C | C | C | C | C | C | C | C | C | C | C | C | C | C | C | T | C | C | C | C | C | C | C | C |
| 166 | G | G | G | G | G | G | G | A | A | A | G | G | G | G | A | C | C | C | C | C | A | C | A | A | A | C | C | C | C | A | A | A | A | C | C | C | A |
| 182 | C | C | C | C | C | T | T | C | C | C | T | C | C | C | C | C | C | C | C | C | C | C | C | C | C | C | C | T | C | C | C | C | C | C | T | T | T |
| 216 | T | T | T | C | T | C | C | C | C | C | C | C | C | C | C | C | C | T | C | T | C | T | C | C | C | T | C | C | T | C | C | C | C | C | C | C | C |
| 222 | T | T | T | T | T | T | T | T | T | T | T | T | T | T | T | T | T | T | T | T | T | T | T | T | T | T | G | T | T | T | T | T | T | T | T | T | T |
| 245 | - | - | - | - | - | - | - | - | - | - | - | - | - | - | - | - | - | - | - | - | - | - | - | - | - | - | - | b | - | - | - | - | - | - | - | - | - |
| 265 | C | C | C | T | C | C | T | C | C | C | C | C | C | C | C | T | C | C | T | C | C | C | C | C | C | C | C | C | C | C | C | C | C | T | C | C | C |
| 282 | C | C | C | C | C | A | C | C | C | C | A | C | C | C | C | C | C | C | C | C | C | C | C | C | C | C | A | A | C | C | C | C | C | C | A | A | A |
| 284 | T | T | T | G | T | G | G | G | G | G | G | G | G | G | G | G | G | T | G | T | G | T | G | G | G | T | G | G | T | G | G | G | G | G | G | G | G |
| 301 | T | T | T | T | T | T | T | T | T | T | T | T | T | T | T | T | T | T | T | T | T | T | T | T | T | T | G | T | T | T | T | T | T | T | T | T | T |
| 327 | A | A | A | A | A | A | A | A | A | A | A | A | A | A | A | A | A | A | A | A | A | A | A | A | A | A | - | A | A | A | A | A | A | A | A | A | A |
| 330 | A | A | A | A | A | C | A | A | A | A | C | A | A | A | A | A | A | A | A | A | A | A | A | A | A | A | A | - | A | A | A | A | A | A | C | C | C |
| 331 | c | c | c | c | c | c | c | c | c | c | c | c | c | c | c | c | c | c | c | c | c | c | c | c | c | c | - | c | c | c | c | c | c | c | c | c | c |
| 342 | A | A | A | T | A | A | A | A | A | A | A | A | A | A | A | A | A | A | A | A | A | A | A | A | A | A | - | A | A | A | A | A | A | A | A | A | A |
| 343 | T | T | T | T | T | G | T | T | T | T | G | T | T | T | T | T | T | T | T | T | T | T | T | T | T | T | T | T | G | T | T | T | T | T | T | T | T |
| 350 | C | C | C | C | C | C | C | C | C | C | C | C | C | C | C | C | C | C | C | C | C | C | C | C | C | C | T | C | C | C | C | C | C | C | C | C | C |
| 354 | G | G | G | G | G | G | G | G | G | G | G | G | T | T | G | G | G | G | G | G | T | G | T | T | T | G | G | G | G | T | T | T | T | G | G | G | G |
| 388 | - | - | - | - | - | - | - | - | - | - | - | - | - | - | - | - | - | - | - | - | - | - | - | - | - | - | - | - | c | - | - | - | - | - | G | G | G |
| 389 | d | d | d | d | d | d | d | d | d | d | d | d | d | d | d | - | d | d | - | d | d | d | d | d | d | d | d | d | d | d | d | d | d | d | d | d | d |
| 398 | e | e | e | e | e | - | e | e | e | e | - | e | e | e | e | e | e | e | e | e | e | e | e | e | e | e | - | - | e | e | e | e | e | e | - | - | - |
| 414 | T | T | T | T | T | T | T | T | T | T | T | T | T | T | T | T | T | T | T | T | T | T | T | T | T | T | C | T | T | T | T | T | T | T | T | T | T |
| 420 | T | T | T | T | T | G | T | T | T | T | G | T | T | T | T | T | T | T | T | T | T | T | T | T | T | T | G | G | T | T | T | T | T | T | G | G | G |
| 426 | T | T | T | G | T | T | G | T | T | T | T | T | T | T | T | G | G | T | G | T | T | T | T | T | T | T | T | T | T | T | T | T | T | G | T | T | T |
| 439 | T | T | T | T | T | G | T | T | T | T | G | T | T | T | T | T | T | T | T | T | T | T | T | T | T | T | G | G | T | T | T | T | T | T | G | G | G |
| 449 | - | - | - | - | - | - | - | - | - | - | - | - | f | f | - | - | - | - | - | - | - | - | - | - | - | - | - | - | - | - | - | - | - | - | - | - | - |
| 494 | A | G | A | A | A | A | A | A | A | A | A | A | A | A | A | A | A | A | A | A | A | A | A | A | A | A | A | A | A | A | A | A | A | A | G | G | G |
| 501 | A | G | A | A | A | G | G | A | A | A | A | A | A | A | A | A | A | A | A | A | A | A | A | A | A | G | G | G | A | A | A | A | A | A | G | G | G |
| 592 | G | G | G | G | A | G | G | G | G | G | G | G | G | G | G | G | G | G | G | G | G | G | G | G | G | G | G | G | G | G | G | G | G | G | G | G | G |
| 593 | G | G | G | T | G | G | G | G | G | G | T | G | G | G | G | G | G | G | G | G | G | G | G | G | G | G | G | G | G | G | G | G | G | G | G | G | G |
| 626 | G | G | G | A | G | G | G | G | G | G | A | G | G | G | G | A | A | A | A | G | G | G | G | G | G | G | G | G | G | G | G | G | G | A | G | G | G |
| 638 | - | - | - | - | - | - | - | - | - | - | - | - | - | - | - | - | - | - | - | g | - | - | - | g | - | - | - | - | - | - | - | - | - | - | - | - | - |
| 665 | G | G | G | G | G | G | G | G | G | G | G | G | G | A | G | G | G | G | G | G | G | G | G | G | G | G | G | G | G | G | G | G | G | G | G | G | G |
| 676 | A | - | A | - | A | - | - | A | A | A | - | A | A | A | A | - | - | - | - | A | A | A | A | A | A | - | - | - | A | A | A | A | A | - | - | - | - |
| 706 | C | T | C | C | C | T | T | C | C | C | C | C | C | C | C | C | C | C | C | C | C | C | C | C | C | C | C | T | C | C | C | C | C | C | T | T | T |
| 719 | G | G | G | G | G | G | G | G | G | G | G | G | G | G | G | G | G | G | G | A | G | G | G | A | G | G | G | G | G | G | G | G | G | G | G | G | G |
| 740 | C | C | C | C | C | C | C | C | C | C | C | C | C | C | C | C | C | C | C | C | C | C | C | C | C | T | T | C | C | C | C | C | C | C | C | C | C |
| 742 | G | G | G | G | T | T | G | G | G | G | G | G | G | G | G | G | G | G | G | G | G | G | G | G | G | T | T | G | G | G | G | G | G | G | G | G | G |
| 747 | C | T | C | C | C | T | T | C | C | C | C | C | C | C | C | C | C | C | C | C | C | C | C | C | C | C | C | T | C | C | C | C | C | C | T | T | T |
| 748 | T | T | T | C | T | T | T | T | T | T | C | T | T | T | T | C | C | C | C | T | T | T | T | T | T | T | T | T | T | T | T | T | T | C | T | T | T |
| 749 | G | G | G | G | G | G | G | G | G | G | G | G | G | G | G | G | G | G | G | G | G | G | G | G | G | T | T | G | G | G | G | G | G | G | G | G | G |
| 750 | A | A | A | A | A | A | A | A | G | A | A | A | A | A | A | A | A | A | A | A | A | A | A | A | A | A | A | A | A | A | A | A | A | A | A | A | A |
| 754 | C | C | A | C | C | C | C | C | C | C | C | C | C | C | C | C | C | C | C | C | A | C | C | C | C | C | C | C | C | C | C | A | C | C | C | C | C |
| 756 | h | - | h | h | - | - | - | - | - | h | - | - | - | - | - | - | - | - | - | h | - | h | - | - | - | - | - | - | - | - | - | - | - | - | - | - | - |
| 771 | - | - | - | - | - | - | - | - | - | - | - | - | - | - | - | - | - | - | - | - | - | - | - | - | - | A | A | - | - | - | - | - | - | - | - | - | - |
| 772 | C | C | C | C | C | - | - | C | C | C | C | C | C | C | C | C | C | C | C | C | C | C | C | C | C | C | C | C | C | C | C | C | C | C | C | C | C |
| 783 | G | G | G | G | G | G | G | T | T | G | G | G | G | G | G | G | G | G | G | G | G | G | G | G | G | G | G | G | G | G | G | G | G | G | G | G | G |
| 808 | C | C | C | C | C | C | C | C | C | C | C | C | C | C | C | C | C | C | C | C | C | C | C | C | C | A | A | C | C | C | C | C | C | C | C | C | C |
| 837 | G | G | G | G | G | G | G | G | G | G | G | G | G | G | G | G | G | G | G | G | G | G | G | G | G | A | A | G | G | G | G | G | G | G | G | G | G |
| 851 | T | T | T | T | T | T | T | T | T | T | T | T | T | T | T | T | T | T | T | T | T | T | T | T | T | T | T | T | T | T | T | T | T | T | G | G | G |
| 867 | C | C | C | C | C | C | C | C | C | C | C | C | C | C | C | C | C | C | C | C | C | T | C | C | C | C | C | C | C | C | C | C | C | C | C | C | C |
| 883 | A | C | A | A | A | C | C | A | A | A | A | A | A | A | A | A | A | A | A | A | A | A | A | A | A | A | C | A | A | A | A | A | A | A | C | C | C |
| 888 | A | T | A | A | A | T | T | A | A | A | A | A | A | A | A | A | A | A | A | A | A | A | A | A | T | T | T | A | A | A | A | A | A | A | T | T | T |
| 890 | - | - | - | - | - | - | - | - | - | - | - | - | - | - | - | - | - | - | - | - | - | - | - | - | - | - | A | A | C | C | C | C | C | C | C | C | C |
| 895 | T | T | T | T | T | T | T | T | T | T | T | T | T | T | T | T | T | T | T | T | T | T | T | T | T | T | C | C | T | T | T | T | T | T | T | T | T |
| 911 | - | - | - | - | - | - | - | - | - | - | - | - | - | - | - | - | - | - | - | - | - | - | - | - | - | - | - | - | - | - | - | - | - | - | i | i | i |
| 921 | T | T | T | T | G | T | T | T | T | T | T | T | T | T | T | T | T | T | T | T | T | G | T | T | T | T | T | T | T | T | T | T | T | T | T | T | T |
| 941 | G | A | G | G | G | A | A | G | G | G | G | G | G | G | G | G | G | G | G | G | G | G | G | G | G | G | G | A | G | G | G | G | G | G | A | A | A |
| 964 | C | C | C | C | C | C | C | C | C | C | C | C | C | C | C | C | C | C | C | C | C | C | C | C | C | T | T | C | C | C | C | C | C | C | C | C | C |
| 1006 | T | T | T | G | T | T | T | T | T | T | G | T | T | T | T | G | G | G | G | T | T | T | T | T | T | T | T | T | T | T | T | T | T | G | T | T | T |
| 1032 | - | - | - | - | - | - | - | - | - | j | - | - | j | - | - | - | - | - | - | - | - | - | - | - | - | - | - | - | - | - | - | - | - | - | - | - | - |
| 1046 | C | C | C | C | C | C | C | C | C | C | C | C | C | C | C | C | C | C | C | C | C | C | C | G | C | C | C | C | C | C | C | C | C | C | C | C | C |
| 1083 | A | C | A | A | A | C | C | A | A | A | A | A | A | A | A | A | A | A | A | A | A | A | A | A | A | A | A | C | A | A | A | A | A | A | C | C | C |
| 1091 | A | G | A | A | A | G | G | A | A | A | A | A | A | A | A | A | A | A | A | A | A | A | A | A | A | A | A | A | A | A | A | A | A | A | A | A | A |
| 1115 | - | - | - | - | - | - | - | - | - | - | - | - | - | - | - | - | - | - | - | - | - | - | - | - | - | - | - | - | - | - | - | - | - | - | - | k | - |
| 1146 | G | G | G | G | G | G | G | G | G | G | G | A | G | G | A | G | G | G | G | G | G | G | G | G | G | G | G | G | G | G | G | G | G | G | G | G | G |
| 1148 | C | C | C | C | C | T | T | C | C | C | C | C | C | C | C | C | C | C | C | C | C | C | C | C | C | C | C | C | C | C | C | C | C | C | C | C | C |
| 1173 | T | T | T | T | T | T | T | T | T | T | T | T | T | T | T | T | T | T | T | T | T | T | T | T | T | T | T | T | T | T | T | T | T | T | G | G | G |
| 1179 | G | G | G | G | G | G | G | G | G | G | G | G | G | G | G | G | G | G | A | G | G | G | G | G | G | G | G | G | G | G | G | G | G | G | G | G | G |
| 1184 | C | C | C | C | C | C | C | C | C | T | C | C | C | C | C | C | C | C | C | C | C | C | C | C | C | C | C | C | C | C | C | C | C | C | C | C | C |
| 1194 | A | A | A | A | A | A | A | A | A | A | A | A | A | A | A | T | A | A | A | A | A | A | A | A | A | A | A | A | A | A | A | A | A | A | A | A | A |
| 1201 | T | T | T | T | T | T | T | T | T | T | T | T | T | T | T | T | T | T | T | T | T | T | T | T | T | A | A | T | T | T | T | T | T | T | - | - | - |
| 1205 | G | G | G | G | G | G | G | G | G | G | G | G | G | G | G | G | G | G | G | G | G | G | G | G | G | T | T | G | G | G | G | G | G | G | G | G | G |
| 1209 | G | G | G | G | G | G | G | G | G | G | G | G | G | G | G | G | G | G | G | G | G | G | G | G | G | T | T | G | G | G | G | G | G | G | G | G | G |
| 1210 | A | A | A | A | A | A | A | A | A | A | A | A | A | A | A | A | A | A | A | A | A | A | A | A | A | T | T | A | A | A | A | A | A | A | A | A | A |
| 1211 | A | A | A | A | A | A | A | A | A | A | A | A | A | A | A | A | A | A | A | A | A | A | A | A | A | T | T | A | A | A | A | A | A | A | A | A | A |
| 1249 | C | C | C | C | C | C | C | C | C | C | C | C | C | C | C | C | C | C | C | C | C | C | C | C | C | C | C | C | C | C | C | C | C | C | C | C | T |
| 1264 | A | A | A | C | A | A | A | A | A | A | C | A | A | A | A | A | C | C | C | C | A | A | A | A | A | T | T | A | A | A | A | A | A | C | A | A | A |
| 1271 | A | C | A | A | A | C | C | A | A | A | A | A | A | A | A | A | A | A | A | A | A | A | A | A | A | C | C | C | A | A | A | A | A | A | C | C | C |
| 1293 | G | G | G | T | G | T | T | G | G | G | T | G | G | G | G | T | T | T | T | G | G | G | G | G | G | T | T | T | G | G | G | G | G | T | T | T | T |
| 1326 | A | G | A | A | A | G | G | A | A | A | A | A | A | A | A | A | A | A | A | A | A | A | A | A | A | G | G | G | A | A | A | A | A | A | G | G | G |
| 1359 | T | C | T | T | T | C | C | T | T | T | T | T | T | T | T | T | T | T | T | T | T | T | T | T | T | C | C | C | T | T | T | T | T | T | C | C | C |
| 1361 | A | - | A | A | A | - | - | A | A | A | A | A | A | A | A | A | A | A | A | A | A | A | A | A | A | - | - | - | A | A | A | A | A | A | - | - | - |
| 1378 | G | G | G | G | G | G | G | G | G | G | G | G | G | G | T | G | G | G | G | G | G | G | G | G | G | G | G | G | G | G | G | G | G | G | G | G | G |
| 1387 | G | G | G | G | G | G | G | T | T | T | G | T | T | T | T | G | G | G | G | G | G | G | T | G | T | G | G | G | G | T | T | G | G | G | G | G | G |
| 1407 | T | G | T | T | T | G | G | T | T | T | T | T | T | T | T | T | T | T | T | T | T | T | T | T | T | G | G | G | T | T | T | T | T | T | G | G | G |
| 1410 | C | T | C | C | C | T | T | C | C | C | C | C | C | C | C | C | C | C | C | C | C | C | C | C | C | C | C | T | C | C | C | C | C | C | T | T | T |
| 1425 | A | A | A | A | A | A | A | A | A | A | A | A | A | A | A | A | A | A | A | A | A | A | A | A | A | C | C | A | A | A | A | A | A | A | A | A | A |
| 1430 | G | G | G | T | G | G | G | G | G | G | T | G | G | G | G | T | T | T | T | G | G | G | G | G | G | G | G | G | G | G | G | G | G | T | G | G | G |
| 1454 | G | G | G | G | G | G | G | G | G | G | G | G | G | G | G | G | G | G | G | G | G | G | G | G | G | G | G | G | G | G | T | G | G | G | G | G | G |
| 1455 | A | A | A | A | A | A | A | A | A | A | A | A | A | A | A | T | A | A | A | A | A | A | A | A | A | A | A | A | A | A | A | A | A | A | A | A | A |
| 1458 | G | G | G | G | G | G | G | G | G | G | G | G | G | G | G | G | G | G | G | G | G | T | G | G | G | G | G | G | G | G | G | G | G | G | G | G | G |
| 1485 | T | T | T | G | T | T | T | T | T | T | G | T | T | T | T | G | G | G | G | T | T | T | T | T | T | G | G | T | T | T | T | T | T | G | T | T | T |
| 1502 | T | G | T | T | T | G | G | T | T | T | T | T | T | T | T | T | T | T | T | T | T | T | T | T | T | T | T | G | T | T | T | T | T | T | G | G | G |

a=AGTATTTTCTTTAGT; b= CCCTTTTCCCGTTTATAT; c= AAATTTTTATTA; d= TTTT; e= TTTTTTTTTTTTTTTTTT; f=

AATAAATACTA; g= ACTTT; h= AAAAAAA; i= GCAGA; j= ATGAAA; k= TATAACTAAT;
