# Supplementary material for: Phylogeography of Parasyncalathium souliei (Asteraceae) and Its Potential Application in Delimiting Phylogeoregions in the Qinghai-Tibet Plateau (QTP)-Hengduan Mountains (HDM) Hotspot
Source: Front Genet. 2018 May 17;9:171. doi: 10.3389/fgene.2018.00171 (PMC5966570; doi:10.3389/fgene.2018.00171)
Supplement: Appendix 3 — Genetic diversity within populations and correlations test. Genetic diversity within populations. (Hd indicates haplotype diversity; π indicates nucleotide diversity). [file Table_3.docx]

Supplementary Material

**Phylogeography of *Parasyncalathium souliei* (Asteraceae) and** **its potential application in delimiting phylogeoregions in the Qinghai-Tibet Plateau (QTP) - Hengduan Mountains (HDM) hotspot**

**Nan Lin^1,2,3#^, Tao Deng^3#^, Michael J. Moore^4^, Yanxia Sun^1^, Xianhan Huang^3^, Wenguang Sun^3^, Dong Luo^3^, Hengchang Wang^1,*^, Jianwen Zhang^3,*^, Hang Sun^3,*^**

^1^Key Laboratory of Plant Germplasm Enhancement and Specialty Agriculture, Wuhan Botanical Garden, Chinese Academy of Sciences, Wuhan, Hubei, China

^2^University of Chinese Academy of Sciences, Beijing, China

^3^Key Laboratory for Plant Diversity and Biogeography of East Asia, Kunming Institute of Botany, Chinese Academy of Sciences, Kunming, Yunnan, China;

^4^Department of Biology, Oberlin College, Oberlin, Ohio, USA;

^#^ These authors have contributed equally to this work.

**^*^ Correspondence:**

Hang Sun, [sunhang@mail.kib.ac.cn](mailto:sunhang@mail.kib.ac.cn);

Jianwen Zhang, [zhangjianwen@mail.kib.ac.cn](mailto:zhangjianwen@mail.kib.ac.cn);

Hengchang Wang, [hcwang@wbgcas.cn](mailto:hcwang@wbgcas.cn)

Appendix 3. Genetic diversity within populations and correlations test.

Genetic diversity within populations. (*Hd* indicates haplotype diversity; π indicates nucleotide diversity)

| Populations | Sample | *Hd* | *π* |
| --- | --- | --- | --- |
| AD | 9 | - | - |
| AL | 16 | 0.242 | 0.00273 |
| AW | 14 | - | - |
| AZ | 12 | - | - |
| BM | 13 | 0.154 | 0.00011 |
| BW | 11 | - | - |
| CN | 12 | - | - |
| CP | 5 | - | - |
| DD | 18 | 0.569 | 0.00997 |
| DX | 16 | 0.342 | 0.00070 |
| GB | 18 | 0.529 | 0.00038 |
| GE | 13 | 0.769 | 0.00987 |
| GJX | 8 | - | - |
| HS | 17 | 0.691 | 0.00141 |
| JC | 19 | 0.579 | 0.00078 |
| JZ | 14 | 0.363 | 0.00026 |
| KS | 4 | 0.000 | - |
| KW | 3 | 0.667 | 0.00189 |
| LDX | 12 | - | - |
| LTE | 17 | - | - |
| LTX | 10 | - | - |
| LW | 17 | 0.515 | 0.00109 |
| ML | 9 | 0.667 | 0.00071 |
| MN | 9 | - | - |
| MX | 10 | 0.200 | 0.00199 |
| QE | 8 | - | - |
| RW | 10 | 0.200 | 0.00014 |
| SJ | 13 | 0.282 | 0.00224 |
| TT | 9 | 0.500 | 0.00893 |
| XL | 13 | 0.615 | 0.00049 |
| YJ | 7 | - | - |
| YL | 9 | 0.750 | 0.00079 |
| ZD | 7 | 0.476 | 0.00034 |
| ZL | 6 | 0.600 | 0.00043 |
| ZM | 10 | 0.600 | 0.00052 |
| ZX | 19 | 0.380 | 0.00718 |


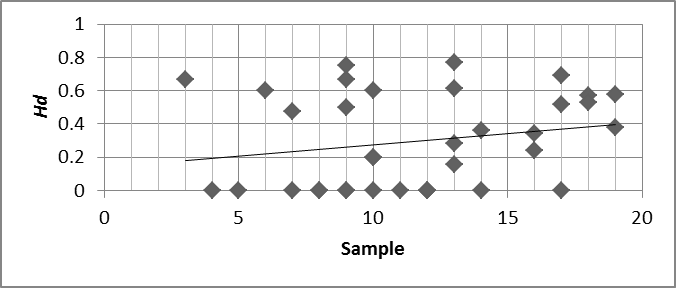


Fig.a Linear regression analysis between the number of sample and haplotype diversity (*Hd* ) of 36 *P. souliei* populations. (r=0.2098, P=0.2194>0.05, haplotype diversity (*Hd* ) is not significant correlated with number of sample).


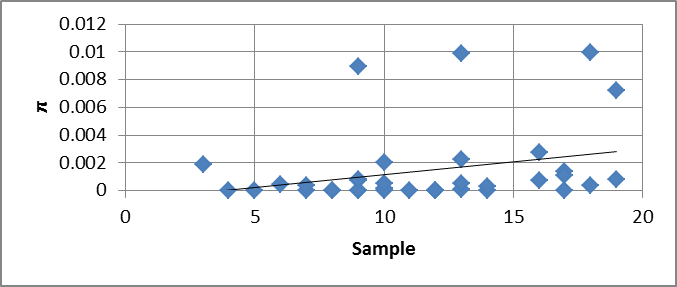


Fig. b. Linear regression analysis between the number of sample and nucleotide diversity (π) of 36 *P. souliei* populations. (r=0.284, P=0.0921>0.05, nucleotide diversity (π) is not significant correlated with number of sample).
