# Supplementary material for: Phylogeography of Parasyncalathium souliei (Asteraceae) and Its Potential Application in Delimiting Phylogeoregions in the Qinghai-Tibet Plateau (QTP)-Hengduan Mountains (HDM) Hotspot
Source: Front Genet. 2018 May 17;9:171. doi: 10.3389/fgene.2018.00171 (PMC5966570; doi:10.3389/fgene.2018.00171)
Supplement: Appendix 5 — Table and Figure of relationship between the fixation index (FCT; y-axis) and number of clusters (K; x-axis) in the SAMOVA of P. souliei. [file Table_5.docx]

Supplementary Material

**Phylogeography of *Parasyncalathium souliei* (Asteraceae) and** **its potential application in delimiting phylogeoregions in the Qinghai-Tibet Plateau (QTP) - Hengduan Mountains (HDM) hotspot**

**Nan Lin^1,2,3#^, Tao Deng^3#^, Michael J. Moore^4^, Yanxia Sun^1^, Xianhan Huang^3^, Wenguang Sun^3^, Dong Luo^3^, Hengchang Wang^1,*^, Jianwen Zhang^3,*^, Hang Sun^3,*^**

^1^Key Laboratory of Plant Germplasm Enhancement and Specialty Agriculture, Wuhan Botanical Garden, Chinese Academy of Sciences, Wuhan, Hubei, China

^2^University of Chinese Academy of Sciences, Beijing, China

^3^Key Laboratory for Plant Diversity and Biogeography of East Asia, Kunming Institute of Botany, Chinese Academy of Sciences, Kunming, Yunnan, China;

^4^Department of Biology, Oberlin College, Oberlin, Ohio, USA;

^#^ These authors have contributed equally to this work.

**^*^ Correspondence:**

Hang Sun, [sunhang@mail.kib.ac.cn](mailto:sunhang@mail.kib.ac.cn);

Jianwen Zhang, [zhangjianwen@mail.kib.ac.cn](mailto:zhangjianwen@mail.kib.ac.cn);

Hengchang Wang, [hcwang@wbgcas.cn](mailto:hcwang@wbgcas.cn)

Appendix 5. Table and Figure of relationship between the fixation index (*F*_CT_; *y*-axis) and number of clusters (*K*; *x*-axis) in the SAMOVA of *P. souliei*.

| *K* | *F*_CT_ |
| --- | --- |
| 3 | 0.67624 |
| 4 | 0.73681 |
| 5 | 0.67513 |
| 6 | 0.72329 |
| 7 | 0.74579 |
| 8 | 0.71193 |
| 9 | 0.75139 |
| 10 | 0.74026 |
| 11 | 0.76131 |
| 12 | 0.7522 |
| 13 | 0.76713 |
| 14 | 0.74805 |
| 15 | 0.75817 |
| 16 | 0.76546 |
| 17 | 0.76923 |
| 18 | 0.75969 |
| 19 | 0.74526 |
| 20 | 0.76323 |


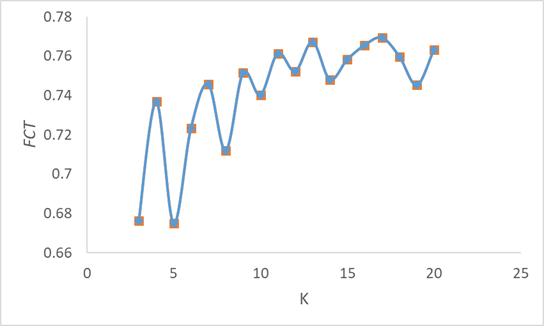


(SAMOVA failed to reveal any meaningful spatial population structure)
