# Supplementary material for: Phylogeography of Parasyncalathium souliei (Asteraceae) and Its Potential Application in Delimiting Phylogeoregions in the Qinghai-Tibet Plateau (QTP)-Hengduan Mountains (HDM) Hotspot
Source: Front Genet. 2018 May 17;9:171. doi: 10.3389/fgene.2018.00171 (PMC5966570; doi:10.3389/fgene.2018.00171)
Supplement: Appendix 6 — Several cases of biogeographic regionalization based on one species from Qinghai-Tibet Plateau (QTP) - Hengduan Mountains (HDM) region. [file Table_6.docx]

Supplementary Material

**Phylogeography of *Parasyncalathium souliei* (Asteraceae) and** **its potential application in delimiting phylogeoregions in the Qinghai-Tibet Plateau (QTP) - Hengduan Mountains (HDM) hotspot**

**Nan Lin^1,2,3#^, Tao Deng^3#^, Michael J. Moore^4^, Yanxia Sun^1^, Xianhan Huang^3^, Wenguang Sun^3^, Dong Luo^3^, Hengchang Wang^1,*^, Jianwen Zhang^3,*^, Hang Sun^3,*^**

^1^Key Laboratory of Plant Germplasm Enhancement and Specialty Agriculture, Wuhan Botanical Garden, Chinese Academy of Sciences, Wuhan, Hubei, China

^2^University of Chinese Academy of Sciences, Beijing, China

^3^Key Laboratory for Plant Diversity and Biogeography of East Asia, Kunming Institute of Botany, Chinese Academy of Sciences, Kunming, Yunnan, China;

^4^Department of Biology, Oberlin College, Oberlin, Ohio, USA;

^#^ These authors have contributed equally to this work.

**^*^ Correspondence:**

Hang Sun, [sunhang@mail.kib.ac.cn](mailto:sunhang@mail.kib.ac.cn);

Jianwen Zhang, [zhangjianwen@mail.kib.ac.cn](mailto:zhangjianwen@mail.kib.ac.cn);

Hengchang Wang, [hcwang@wbgcas.cn](mailto:hcwang@wbgcas.cn)

Appendix 6. Several cases of biogeographic regionalization based on one species from Qinghai-Tibet Plateau (QTP) - Hengduan Mountains (HDM) region.

| Species | Animals or plants | Marker | Location range | Units in floristics of Wu and Wu(1996) | Putative biogeographic boundaries | Reference |
| --- | --- | --- | --- | --- | --- | --- |
| *Babina pleuraden* Boulenger | animal | *COI*, *cyt* *b* (mitochondrial) | 24°~26° N  98°~105° E | IIID10d, IIIE14a, IIIE14b, IIIE13a, IIIE13b, IIIE13c. | Jingsha River Line | (Li et al., 2012) |
| *Cupressus* L*.* | plant | *trnD-trnT, trnS-trnG and trnL-trnF* (chloroplast) | 24°~33° N  82°~102° E | IIIE13a, IIIE14a, IIIE14b, IIIE14c, IIIE15b, IIIE16b, IIIE16c, IIIE17a, IIIE17b. | Hengduan Mountains -Yungui Plateau | (Xu et al., 2010) |
| *Oxyria sinensis* Hemsl. | plant | *matK*(chloroplast) | 25°~32° N  95°~105° E | IID10a,IIID10d,IIIE14a, IIIE14b, IIIE14c，IIIE15b. | Mekong–Salween Line | (Meng et al., 2015) |
| *Sinopodophyllum hexandrum* (Royle) Ying | plant | *atpH–atpI, rps18–clpp, rpl32trnL*(chloroplast) | 28°~32° N  93°~108° E | IIID10a,IIID10d,IIIE14a, IIIE14b, IIIE14c，IIIE15b. | Mekong–Salween Line | (Li et al., 2011) |
| *Taxus wallichiana* Zucc. | plant | *trnL*-*trnF* (chloroplast) | 25°~29° N  97°~103° E | IIIE13a, IIIE14a, IIIE14b, IIIE14c. | Mekong–Salween Line | (Liu et al., 2013) |
| *Terminalia franchetii* [Gagnep.](https://www.baidu.com/link?url=UfHWpG7d3_Wrx4nw9Whuaac5ZbptD8IgvpF0v36LDSBVr7s5Fr0O-maWcp_OE8Kf6R4o9RTLrMn2wydt6dYlBq&wd=&eqid=a5dc6d7200018671000000035a9ff727) | plant | *trnL–F*, *petL–psbE* (chloroplast) | 23°~30° N  98°~105° E | IIIE13a, IIIE14a, IIIE14b, IIIE14c，IIIE15b. | Jingsha River Line | (Zhang et al., 2011) |
| *Populus davidiana-rotundifolia* | plant | *matK, trnG-psbK, psbK-psbI, and ndhC-trnV* (chloroplast); SSR(nuclear) | 20°~50° N  85°~135° E | IIIE13a, IIIE14a, IIIE14b, IIIE14c，IIIE15b, IIIE16a,IIIE17a, IIID8a, IIID8B, IIID8c, IB4a,IIID7. | Jingsha River Line | (Zheng et al., 2017) |
| *Quercus aquifolioides* [Rehd. et Wils.](https://www.baidu.com/link?url=RMeu7CzBXKCk98OHCWXG_WtVimqgvUf3jR1jp6Wqs9x16PFddtvsFE3wuhJMZNU0-3oAJj3X69443m5DB5SWq_&wd=&eqid=a419eac7000200d0000000035ab07d1c) | plant | *trnH-psbA, rpS16, trnS - trnT trnQ- trnS* (chloroplast); SSR(nuclear) | 25°~35° N  95°~105° E | IID10a,IIID10d,IIIE14a, IIIE14b, IIIE14c，IIIE15b. | Salween Line | (Du et al., 2017) |

References

Du, F.K., Hou, M., Wang, W., Mao, K., and Hampe, A. (2017). Phylogeography of *Quercus aquifolioides* provides novel insights into the Neogene history of a major global hotspot of plant diversity in south-west China. *Journal of Biogeography* 44**,** 294-307. doi: 10.1111/jbi.12836.

Li, Y., Zhai, S.N., Qiu, Y.X., Guo, Y.P., Ge, X.J., and Comes, H.P. (2011). Glacial survival east and west of the 'Mekong-Salween Divide' in the Himalaya-Hengduan Mountains region as revealed by AFLPs and cpDNA sequence variation in *Sinopodophyllum hexandrum* (Berberidaceae). *Molecular Phylogenetics and Evolution* 59(2)**,** 412-424. doi: 10.1016/j.ympev.2011.01.009.

Li, Z., Yu, G., Rao, D., and Yang, J. (2012). Phylogeography and demographic history of *Babina pleuraden* (Anura, Ranidae) in southwestern China. *PLoS One* 7(3)**,** e34013. doi: 10.1371/journal.pone.0034013.

Liu, J., Moller, M., Provan, J., Gao, L.M., Poudel, R.C., and Li, D.Z. (2013). Geological and ecological factors drive cryptic speciation of yews in a biodiversity hotspot. *New Phytologist* 199**,** 1093-1108. doi: 10.1111/nph.12336.

Meng, L., Chen, G., Li, Z., Yang, Y., Wang, Z., and Wang, L. (2015). Refugial isolation and range expansions drive the genetic structure of *Oxyria sinensis* (Polygonaceae) in the Himalaya-Hengduan Mountains. *Scientific Report* 5**,** 10396. doi: 10.1038/srep10396.

Wu, Z.Y., and Wu, S.G. (1996). A proposal for a new floristic kingdom (realm): the East Asiatic Kingdom, its delineation and characteristics. Floristic characteristics and diversity of East Asian plants, proceedings of the first international symposium on floristic characteristics and diversity of East Asian plants (ed. by A.L. Zhang and S.G. Wu). *China Higher Education Press, Beijing.***,** pp. 3-42.

Xu, T., Abbott, R.J., Milne, R.I., Mao, K., Du, F.K., Wu, G., et al. (2010). Phylogeography and allopatric divergence of cypress species (*Cupressus* L.) in the Qinghai-Tibetan Plateau and adjacent regions. *BMC Evolution Biology* 10**,** 194. doi: 10.1186/1471-2148-10-194.

Zhang, T.C., Comes, H.P., and Sun, H. (2011). Chloroplast phylogeography of *Terminalia franchetii* (Combretaceae) from the eastern Sino-Himalayan region and its correlation with historical river capture events. *Molecular Phylogenetics and Evolution* 60**,** 1-12. doi: 10.1016/j.ympev.2011.04.009.

Zheng, H., Fan, L., Milne, R.I., Zhang, L., Wang, Y., and Mao, K. (2017). Species delimitation and lineage separation history of a species complex of aspens in China. *Frontiers in Plant Science* 8**,** 375. doi: 10.3389/fpls.2017.00375.


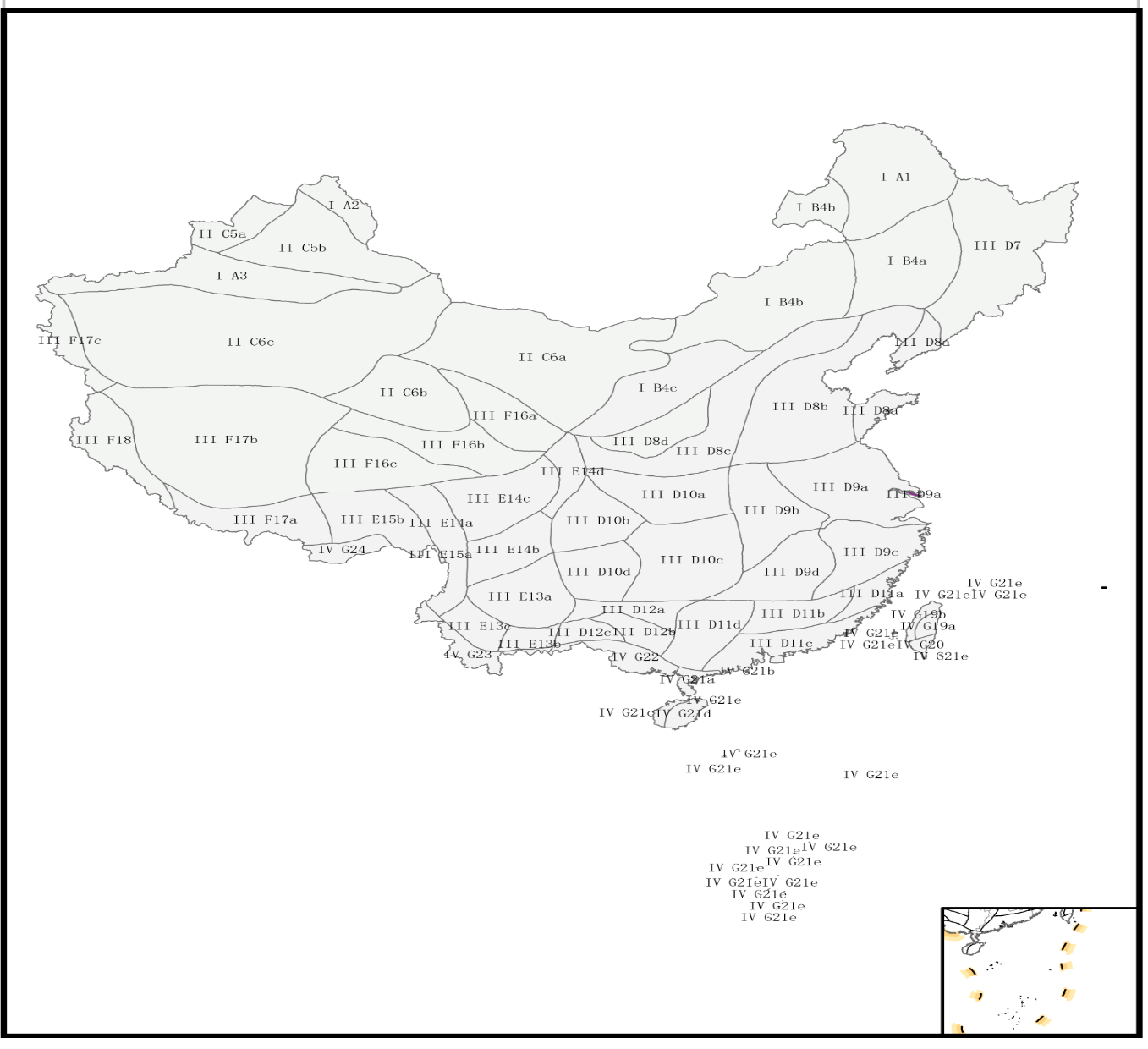


Note: The divisions of China based on Wu and Wu (1996), the Roman numbers represent: I, Holarctic Kingdom; II, Tethys Kingdom; III, East Asiatic Kingdom; IV, Paleotropic Kingdom.
